# Preclinical assessment of synergistic efficacy of MELK and CDK inhibitors in adrenocortical cancer

**DOI:** 10.1186/s13046-022-02464-5

**Published:** 2022-09-23

**Authors:** Dipranjan Laha, Robert R.C. Grant, Prachi Mishra, Myriem Boufraqech, Min Shen, Ya-Qin Zhang, Matthew D. Hall, Martha Quezado, Michelly Sampaio De Melo, Jaydira Del Rivero, Martha Zeiger, Naris Nilubol

**Affiliations:** 1 Surgical Oncology Program, Center for Cancer Research, National Cancer Institute, National Institutes of Health, Bethesda, MD 20892 USA; 2 Department of Molecular Biosciences, College of Natural Sciences, Institute for Cellular and Molecular Biology, University of Texas at Austin, Austin, TX USA; 3 National Center for Advancing Translational Sciences, National Institutes of Health, Rockville, MD USA; 4 Laboratory of Pathology, Center for Cancer Research, National Cancer Institute, National Institutes of Health, Bethesda, MD USA; 5 Developmental Therapeutics Branch, Center for Cancer Research, National Cancer Institute, National Institutes of Health, Bethesda, MD USA

**Keywords:** Adrenocortical cancer, Maternal embryonic leucine zipper kinase, Cyclin-dependent kinase, β-catenin, Quantitative high-throughput screening, Targeted therapy

## Abstract

**Background:**

Adrenocortical cancer (ACC) is a rare and aggressive cancer with dismal 5-year survival due to a lack of effective treatments. We aimed to identify a new effective combination of drugs and investigated their synergistic efficacy in ACC preclinical models.

**Methods:**

A quantitative high-throughput drug screening of 4,991 compounds was performed on two ACC cell lines, SW13 and NCI-H295R, based on antiproliferative effect and caspase-3/7 activity. The top candidate drugs were pairwise combined to identify the most potent combinations. The synergistic efficacy of the selected inhibitors was tested on tumorigenic phenotypes, such as cell proliferation, migration, invasion, spheroid formation, and clonogenicity, with appropriate mechanistic validation by cell cycle and apoptotic assays and protein expression of the involved molecules. We tested the efficacy of the drug combination in mice with luciferase-tagged human ACC xenografts. To study the mRNA expression of target molecules in ACC and their clinical correlations, we analyzed the Gene Expression Omnibus and The Cancer Genome Atlas.

**Results:**

We chose the maternal embryonic leucine zipper kinase (MELK) inhibitor (OTS167) and cyclin-dependent kinase (CDK) inhibitor (RGB-286638) because of their potent synergy from the pairwise drug combination matrices derived from the top 30 single drugs. Multiple publicly available databases demonstrated overexpression of MELK, CDK1/2, and partnering cyclins mRNA in ACC, which were independently associated with mortality and other adverse clinical features. The drug combination demonstrated a synergistic antiproliferative effect on ACC cells. Compared to the single-agent treatment groups, the combination treatment increased G2/M arrest, caspase-dependent apoptosis, reduced cyclins A2, B1, B2, and E2 expression, and decreased cell migration and invasion with reduced vimentin. Moreover, the combination effectively decreased Foxhead Box M1, Axin2, glycogen synthase kinase 3-beta, and β-catenin. A reduction in p-stathmin from the combination treatment destabilized microtubule assembly by tubulin depolymerization. The drug combination treatment in mice with human ACC xenografts resulted in a significantly lower tumor burden than those treated with single-agents and vehicle control groups.

**Conclusions:**

Our preclinical study revealed a novel synergistic combination of OTS167 and RGB-286638 in ACC that effectively targets multiple molecules associated with ACC aggressiveness. A phase Ib/II clinical trial in patients with advanced ACC is therefore warranted.

**Supplementary Information:**

The online version contains supplementary material available at 10.1186/s13046-022-02464-5.

## Background

ACC is a rare and aggressive endocrine malignancy with a yearly incidence of 0.5–2 cases per million population [1, 2]. Due to late clinical presentation and the aggressive behavior of this tumor, patients with ACC commonly present at an advanced stage [2–4]. Unfortunately, loco-regional recurrence and distant metastasis are common (up to 50–80%) despite complete surgical resection [5, 6]. The high mortality rate in patients with advanced ACC is due to aggressive biology and a lack of effective systemic treatments. In the past five decades, mitotane has been the only FDA-approved treatment in patients with advanced ACC and is first-line systemic therapy alone or with etoposide, doxorubicin, and cisplatin, a poorly tolerated and minimally efficacious regimen based on the FIRM-ACT trial [7]. Overall survival (OS) remains poor with these therapies. Thus, there is a critical need to identify novel, effective treatment strategies. The comprehensive molecular analyses in large cohorts of patients by the European Network for the Study of Adrenal Tumors (ENSAT) and TCGA provide crucial information by identifying important mutations and dysregulated pathways by molecular signatures [8, 9]. Approximately 40% of ACC samples in TCGA and ENSAT cohorts had pathogenic alterations in genes involved in the Wnt/β-catenin signaling pathway and many alterations involving genes that regulate the cell cycle*.* To date, there have been no treatments that effectively target the key downstream molecules of these pathways.

As we previously demonstrated, quantitative high-throughput drug screening (qHTS) using the pharmaceutical library containing agents tested or used in humans is an effective and efficient way to identify single-drug candidates and drug combinations that could be readily translated to a phase Ib/II clinical trial in patients with advanced ACC [10]. In this study, the combination of the maternal embryonic leucine zipper kinase (MELK) inhibitor (OTS167) and the cyclin-dependent kinase **(**CDK) inhibitor (RGB-286638) was identified as a novel treatment in ACC. We validated the synergy of these drugs in multiple in vitro models and comprehensively elucidated their mechanisms via the inhibition of multiple key downstream molecules associated with the Wnt/β-catenin signaling pathway and the induction of G2/M cell cycle arrest. Combination treatment inhibited cell invasion and reduced the epithelial-to-mesenchymal transition (EMT) markers. Furthermore, we confirmed in in vivo systems that mice treated with OTS167 and RGB-286638 combination had a significantly lower tumor burden than those in control groups. These results demonstrate the effectiveness of these agents against ACC in preclinical studies and support a further evaluation in a clinical trial.

## Materials and methods

### Patient samples

Human adrenocortical tissue samples were collected under the clinical protocol entitled “Prospective comprehensive molecular analysis of endocrine neoplasms” (Clinical Trial Registration number NCT01005654). The ethical approval was granted by the Institutional Review Board, National Cancer Institute, NIH, and the NIH Office of Human Subject Research. All participants provided written informed consent.

### qHTS and combination matrix screening

The National Center for Advancing Translational Sciences (NCATS) Pharmaceutical Collection (NPC) and the Mechanism Interrogation PlatE (MIPE) library, which in total consist of 4,991 small-molecule drugs and investigational compounds, were screened against SW13 and NCI-H295R cell lines. Cell viability was measured using a luciferase-coupled ATP quantitation assay (CellTiter-Glo®, Promega, Madison, WI). We described the detailed methods of quantitative high-throughput screening in the supplementary section.

A combination matrix screen was performed with a subset of active hits identified by qHTS. Plating of compounds in matrix format using acoustic droplet ejection and numerical characterization of synergy, additivity, and/or antagonism was conducted as described previously [11, 12]. The detailed method of combination matrix screening was described in the supplementary section.

### Gene expression profiling

We analyzed publicly available genome-wide expression data from the Gene Expression Omnibus (GEO) (NCBI gene expression and hybridization array data repository) in three cohorts (GSE33371, GSE12368, and GSE90713) to study the differential messenger RNA (mRNA) expression of genes of interest in human ACC samples compared to adrenocortical adenoma (ACA) and normal adrenal cortex (NC) [13]. Using data from TCGA and the European Bioinformatics Institute (E-TABM-311), we analyzed clinicopathologic correlations with mRNA expression of these genes to assess clinical relevance.

### Immunohistochemistry analysis

ACC, ACA patient tissue, and human ACC xenograft tissues were formalin-fixed, embedded in paraffin, and used for immunohistochemistry (IHC) analysis. 5-µm-thick sections were used for hematoxylin and eosin (H&E) and IHC staining according to a previously published protocol [10]. We described the IHC techniques in the supplementary materials section.

### Cell lines and culture conditions

Two human ACC cell lines, SW13 and NCI-H295R, were purchased from the American Type Culture Collection™ (Cat # CCL-105, CRL-2128; Manassas, VA, USA) and cultured in 5% CO_2_ atmosphere at 37 °C in Dulbecco's Modified Eagle Medium (Cat # 11195–065, Thermo Fisher Scientific, MA, USA) supplemented with 2.5% Nu-Serum (Cat # 355100, Corning, MA, USA) and 0.1% Insulin-Transferrin-Selenium (Cat # 41400045, Thermo Fisher Scientific, MA, USA). Cell lines were authenticated by short tandem repeat profiling. We routinely subcultured every 3–5 days, depending on the degree of cell confluence.

NCI-H295R cells used to generate human ACC xenograft were transfected with a linearized pGL4.51[*luc2*/CMV/Neo] vector (9PIE132, Promega) encoding the luciferase reporter gene *luc2* (*Photinus pyralis*) and maintained in the above medium with up to 500 μg/mL of G-418 antibiotic (11811–023, Gibco, MA, USA) for selection.

### Cellular proliferation assay

The effects of drug treatments on cell proliferation were quantitated by CyQuant assay (Cat # C7026, Invitrogen, MA, USA). SW13 (3 × 10^3^) and NCI-H295R (6 × 10^3^) cells were plated in 96-well black plates (Cat # 353219, Costar®, Corning, NY, USA). After 24 h, the culture medium containing vehicle control (dimethyl sulfoxide up to 0.125%), OTS167, RGB-286638, or the combination of OTS167 and RGB-286638 were added at various concentrations. The medium with the drugs or vehicle was replaced every 48 h. Fluorescence intensity was determined using a microplate reader (Molecular Devices, Sunnyvale, CA, USA) at 485 nm/538 nm. We repeated the experiment with consistent results at least three times.

We used the automated computerized algorithm (Chou–Talalay method) to assess the synergistic efficacy. Efficacy indicated by the combination index (CI) was compared to cells treated with a single drug. CI < 1 indicated synergy; CI = 1 indicated an additive effect; and CI > 1, indicated an antagonistic effect [14].

### Three-dimensional multicellular aggregates (MCA)

Compared to monolayer cell culture that lacks the tumor microenvironment, MCAs recapitulate the in vivo environment by growing solid, 3-dimensional tumors in vitro more accurately as MCAs contain different areas affected by various degrees of oxygenation, nutrients, and drug exposure [15]. SW13 (6 × 10^4^ cells/0.5 ml) and NCI-H295R (1 × 10^5^ cells/0.5 ml) cells, which form multicellular aggregates (MCA) or tumor spheroids, were plated in ultra-low cluster 24-well plates (Cat No # 3473, Costar®, Corning, NY, USA). The anticancer activity of OTS167, RGB-286638, and the combination of OTS167 and RGB-286638 were tested in MCAs that mimic solid tumors in vitro, in ACC cell lines according to standard protocol. The detailed methods are described in the supplementary materials section.

### Clonogenic assay

Cells were seeded in triplicate in 6-well plates (1000 cells/well) and allowed to grow for 7–10 days. The cells were then treated with drug(s) alone or in combination or with the vehicle in complete media for 12 to 14 days. Growth media with vehicle or drug(s) were replaced every 48–72 h. The cells were fixed with 0.4% buffered paraformaldehyde and then stained with 0.5% crystal violet in methanol for 10 min. The colonies were counted and photographed using a ChemiDoc system (Bio-Rad).

### Caspase-3/-7 activation assay

To check caspase-3/7-mediated apoptosis, cells were plated in 96-well plates and treated for 24 to 48 h with various concentrations of the drug combination. The caspase-3/-7 activity was measured using the Caspase-Glo® 3/7 assay (Cat # G8091, Promega, USA), according to the manufacturer's instructions. The method of treatment for caspase-3 and -7 activation and analysis are described in the supplementary materials section.

### Cell cycle assay

SW13 (3 × 10^4^) and NCI-H295R (2 × 10^5^) cells were plated in a 100-mm dish with 10 mL of culture medium and treated 24 h later. After 24 to72 hours, cells were trypsinized, washed with phosphate-buffered saline (PBS), and fixed with ice-cold 70% ethanol. Cells were then resuspended in PBS with ribonuclease A (100 mg/mL) and propidium iodide (PI) (0.05 mg/mL) for fluorescence-activated cell sorting analysis using the Canto II flow cytometer (Becton Dickinson, Franklin Lakes, NJ, USA). Data were generated for at least 20,000 events per sample. The cell cycle of the gated PI distribution was analyzed using ModFit software (Verity Software House, Inc., Topsham, ME, USA).

### Western blot analysis

Cell lysates were prepared from the cells after treatment with drug(s) or vehicle control. The protein concentration was determined using the Pierce™ BCA assay kit solution (Cat # 23227, Thermo Fisher Scientific, Waltham, MA, USA). An equal amount of proteins from different treatment conditions was used for western blot experiments. The western blot techniques are described in the supplementary materials section. Because the treatments directly affected ACC cell cycles and the doubling time of SW13 and NCI-H295R cells are approximately 24 and 48 h, respectively, the optimal time to capture the changes in molecular signaling was shorter in SW13 (24–48 h) than in NCI-H295R (48–72 h) before ACC cells underwent treatment-related apoptosis.

### Cellular migration and invasion assay

To determine the effects of a single drug or combination drugs on the migratory and invasive capacity of ACC cells, we performed cellular migration and invasion assays according to the manufacturer’s instruction (Cat # 354578, Cat # 3544880, BD Bioscience, San Joes, CA, USA). ACC cells were plated in six-well plates in a triplicate manner and treated with varying concentrations of OTS167, RGB-286638, the combination of OTS167 and RGB-286638, and vehicle control for 24 h and 48 h, respectively. The methods of cellular migration and invasion assay are described in the supplementary materials section.

### Immunofluorescence analysis

2 × 10^5^ SW13 and NCI-H295R cells were plated on glass coverslips, allowed to attach overnight, and treated for 48 to 72 h. Cells were fixed with 4% paraformaldehyde, permeabilized in 0.25% Triton-X, and incubated overnight with primary antibodies. DNA was stained with DAPI (Vector Laboratories, Burlingame, CA, USA). Images were obtained by fluorescence microscopy with 40 × magnification and collected using Carl Zeiss ZEN Software (Zeiss, Germany)**.**

### Separation of polymerized and depolymerized tubulin

Tubulin polymerization and depolymerization assay was performed to check the effects of drug treatments on the tubulin polymerization process in ACC cells. Therefore, SW13 (1 × 10^6^) and NCI-H295R (1 × 10^6^) cells were plated in a 100 mm dish with 10 mL of culture medium. Cells were treated with drug(s) and vehicle control for 48 h for SW13 and 72 h for NCI-H295R cells. The method of separation of polymerized and depolymerized tubulin assays is described in the supplementary materials section.

### Oligo small interfering RNA (siRNA)-mediated transfection

SW13 and NCI-H295R cells were transfected with small interfering RNA (siRNA) specific for MELK (4390824, assay ID s386, Thermo Fisher Scientific, MA, USA) or control siRNA (4390843, Thermo Fisher Scientific, MA, USA) using Lipofectamine RNAiMAX (13778–015, Invitrogen; Thermo Fisher Scientific, Inc., MA, USA). After 48 h of transfection, Western Blot was performed to check the transfection efficiency of MELK knockdown and target proteins.

### *In vivo* study

The protocol designed to study the in vivo efficacy of OTS167 and RGB-286638 in mice with human ACC xenografts was approved by the National Cancer Institute, National Institutes of Health (NIH), Animal Care and Use Committee. Mice were maintained according to NIH Animal Research Advisory Committee guidelines. A total of 5 × 10^6^ NCI-H295R cells with luciferase reporter in Corning® Matrigel® Matrix (Cat # 354234, Corning, NY, USA) were injected into each flank of a Nuþ/Nuþ mouse (two xenografts per mouse). After 21 days, mice were randomized into four groups by the treatment. We selected the dosages of OTS167 and RGB-286638 based on prior publications that showed in vivo efficacy with no signs of treatment-related toxicities in mice [16, 17]. Treatments included: Group 1: 0.1% DMSO as vehicle control; Group 2: daily (Monday-Friday) OTS167 (10 mg/kg) via intraperitoneal injection; Group 3: RGB-286638 (20 mg/kg) using an intravenous injection (IV) via tail vein three times (Monday, Wednesday, Friday) weekly for two weeks, followed by RGB-286638 drugs (6 mg/100 μl) loaded ALZET pumps with 0.25 μl/hour delivery rate (Model 1002, Alzet, Cupertino, CA, USA) Group 4: the combination of OTS167 (10 mg/kg) and RGB-286638 (20 mg/kg), following the above protocol for drug administrations. The duration of treatment was five weeks. Mice received daily health monitoring, and mouse weight was recorded weekly. In brief, the details of in vivo imaging studies are described in the supplementary materials.

### Statistical analysis

Statistical analyses were performed using SPSS version 25.0 for Windows (SPSS, Inc., Chicago, IL, USA) and GraphPad Prism 8 software (GraphPad Software, La Jolla, CA, USA). Statistical analysis methods are described in the supplementary materials section.

## Results

### qHTS identified effective combination therapy in ACC

**Fig. 1:**
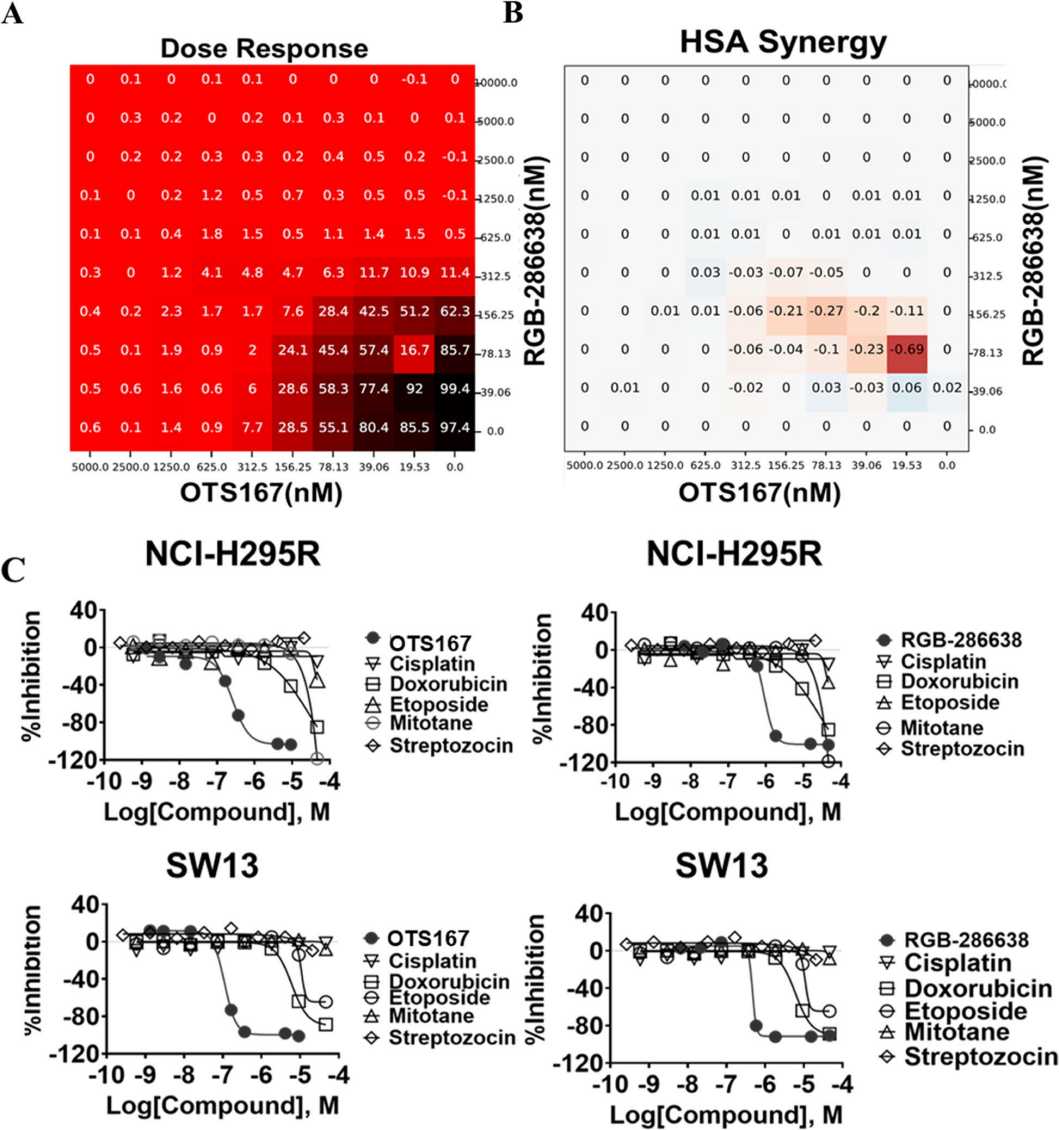
Drug candidates identified through high throughput drug screening in ACC: **A** 10 × 10 combination matrix screening data demonstrating the drug synergy between OTS167 and RGB-286638. **B** HSA synergy of OTS167 and RGB-286638 drugs. **C** Dose–response curve comparing OTS167 (left panel) and RGB-286638 (right panel) with the current standard of care chemotherapy: cisplatin, doxorubicin, etoposide, mitotane, and streptozocin in NCI-H295R and SW13 cells

The results of qHTS produced 85 compounds with efficacy > 80% at concentrations < 1 µM in both cell lines. An orthogonal assay to measure caspase-3/-7 activation identified 30 of these 85 compounds that were then tested in pairwise combination matrices (Supplementary Table 1). The combination of OTS167 and RGB-286638 showed a potent synergistic effect based on the highest single agent (HSA) and Bliss synergy model scores at concentrations in the nanomolar range (Fig. 1A, B). We then compared the single-agent activity of these drugs to current systemic therapy for patients with advanced ACC. Each had higher efficacy at equal or lower concentrations than individual treatment EDP-M or streptozocin in both cell lines (Fig. 1C).

### Expressions of MELK, CDK 1 and 2, and partnering cyclins are associated with poor clinical outcomes in ACC

**Fig. 2:**
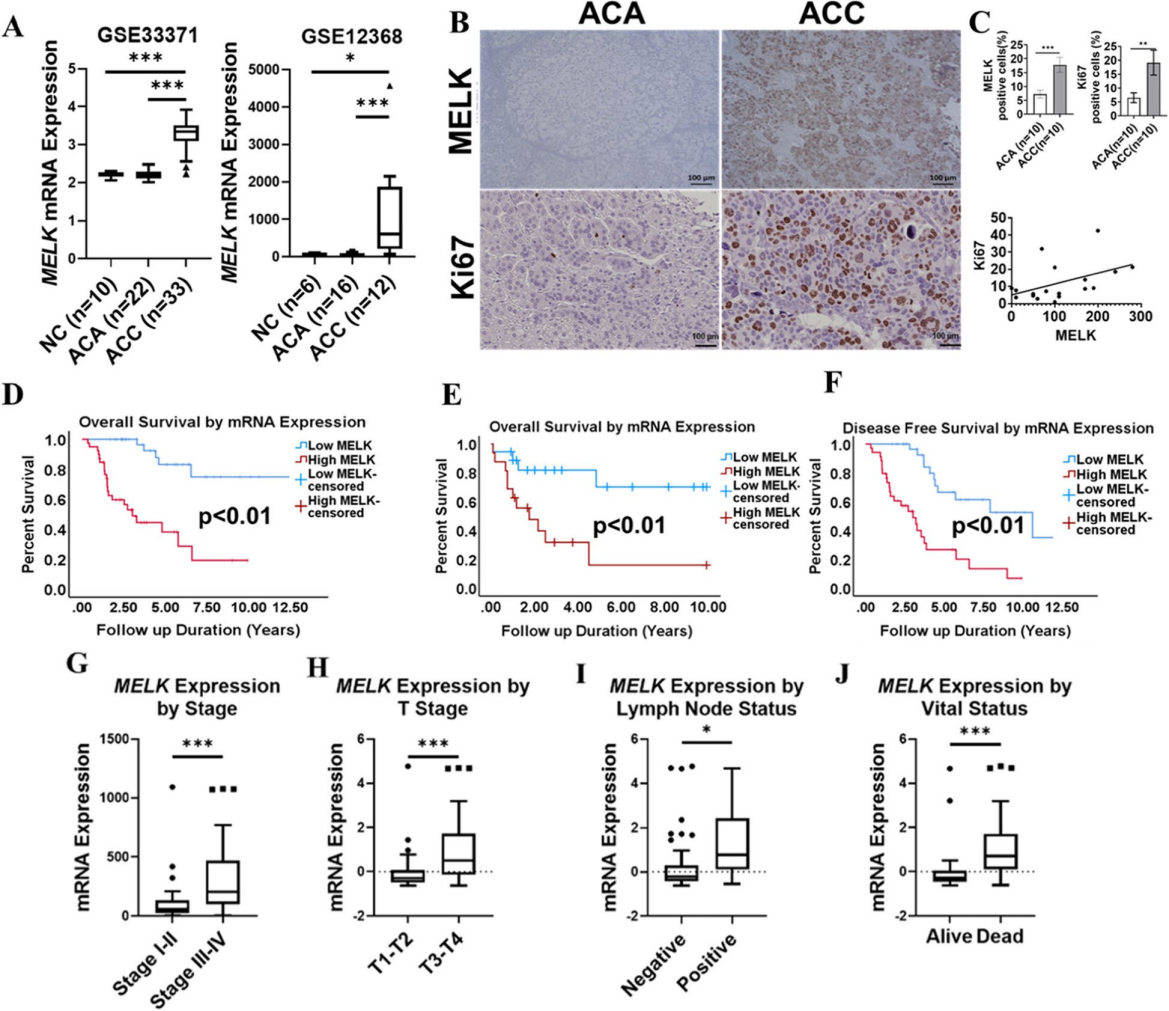
MELK overexpression is associated with poor prognosis in ACC: **A** mRNA expression of *MELK* from publicly available data sets GSE33371 (NC = 10, ACA = 22, and ACC = 33) and GSE12368 (NC = 6, ACA = 16, and ACC = 12), respectively. **B** IHC study for MELK and Ki67 expression in the NIH cohort of ACA (*n* = 10) and ACC (*n* = 10) (*p* < 0.05). **C** Quantification graph of MELK and Ki67 expression in ACC (*p*<0.01) (upper plan); Ki67 and MELK correlation graph from IHC scoring data (*p* = 0.04) (lower plan). **D** Kaplan–Meier survival curve representing the OS in TCGA ACC patients (*n* = 78) with high and low MELK expression. High *MELK* expression (red) (*n* = 39); low *MELK* expression (blue) (*n* = 39) (*p < 0.01). **E** Kaplan–Meier survival curve representing the OS in E-TABM-311 ACC patients (*n* = 34) with high and low *MELK* expression. High *MELK* expression (red) (*n* = 16); low MELK expression (blue) (*n* = 18) (*p < 0.01). **F** Kaplan–Meier survival curve representing the overall DFS in TCGA ACC patients with high and low *MELK* expression. High *MELK* expression (red) (*n* = 16); low *MELK* expression (blue) (*n* = 18) (*p < 0.01). **G** Differential mRNA expression of *MELK* by overall stages (stage I, II [*n* = 46] vs. stage III, IV [*n* = 30]) in the TCGA ACC patient data set (*p < 0.05). **H** Differential mRNA expression of *MELK* by tumor stages in the TCGA ACC patient data set (T1–T2 [*n* = 51] vs. T3–T4 [*n* = 25]) (*p < 0.05). **I** Differential mRNA expression of *MELK* by lymph node status in the TCGA ACC cohort (*p < 0.05). Lymph node positive (*n* = 67); lymph node negative (*n* = 9). **J** Differential mRNA expression of *MELK* by alive (*n* = 51) and dead status (*n* = 27) of ACC patients data in the TCGA cohort (*p < 0.05)

Because OTS167 is a MELK inhibitor, we aimed to determine if ACC overexpresses *MELK* mRNA in human samples. *MELK* mRNA expression was significantly higher in ACC compared to ACA and NC in two GEO cohorts (Fig. 2A). Next, we analyzed MELK protein expression and its relation to Ki67, a marker of proliferation commonly used as a prognostic indicator in ACC, by IHC in human adrenal samples from the NIH cohort and found a linear and, positive correlation (Spearman’s correlation coefficient 0.5049, *p* = 0.04) (Fig. 2B, C) [18, 19]. Analysis of the TCGA and E-TABM-311 databases showed that the estimated overall survival (OS) of patients with ACC that overexpressed *MELK* was shorter than patients with low *MELK* mRNA expression (TCGA: 4.3 years vs. 10.8 years, *p* < 0.001; E-TABM-311: 3.1 years vs. 7.7 years, *p* = 0.004) (Fig. 2D, E). Moreover, in the TCGA cohort, overexpression of *MELK* was associated with shorter disease-free survival (DFS) (3.6 years vs. 8.6 years, *p* < 0.001), more advanced tumor stage and overall stage, lymph node metastasis, and death during follow-up (Fig. 2F-J).

Because RGB-286638 targets the kinase activity of multiple CDKs, we analyzed mRNA expressions of CDK1/2 and their associated cyclins in the GEO cohorts. Previous work has shown overexpression of *CDK1* and *CDK2* in ACC [10]. Here, the additional independent data set (GSE90713) confirmed that *CDK1* mRNA expression, but not *CDK2*, was significantly higher in ACC compared to NC (Supplementary Fig. 1A). We also found a significant positive linear correlation between the mRNA expression of *MKI67* and both *CDK1* and *CDK2* (Supplementary Table 2). Because CDK inhibitors do not change the protein expression of CDKs, and measuring CDK activity as a marker of treatment response is impractical, we studied the expression of the partnering cyclins and their clinical relevance in ACC retrospectively. We found overexpression of cyclin A2 (*CCNA2*)*,* cyclin B1 (*CCNB1)*, cyclin B2 (*CCNB2),* and cyclin E2 (*CCNE2)* mRNA in ACC compared to NC and ACAwith the exception of *CCNE2* in GSE90713 (Supplementary Fig. 1B–E). Next, we confirmed the positive linear correlations between the mRNA expression of *MKI67* vs. *CCNA2, CCNB1, CCNB2,* and *CCNE2*, suggesting their role in tumor progression (Supplementary Table 2). From the TCGA dataset, we confirmed that patients with *CCNA2*, *CCNB1*, *CCNB2*, and *CCNE2* overexpression had significantly shorter OS, DFS, higher overall stage, and higher T stage compared to patients with low mRNA expressions (Supplementary Fig. 2A-D, 3A-C). We also found that *MELK* expression strongly correlated with *CDK1, CDK2, CCNA2, CCNB1, CCNB2,* and *CCNE2* mRNA expressions (Supplementary Table 3).

### OTS167 and RGB-286638 combination treatment synergistically inhibits cell proliferation, MCAs, and colony formation

**Fig. 3:**
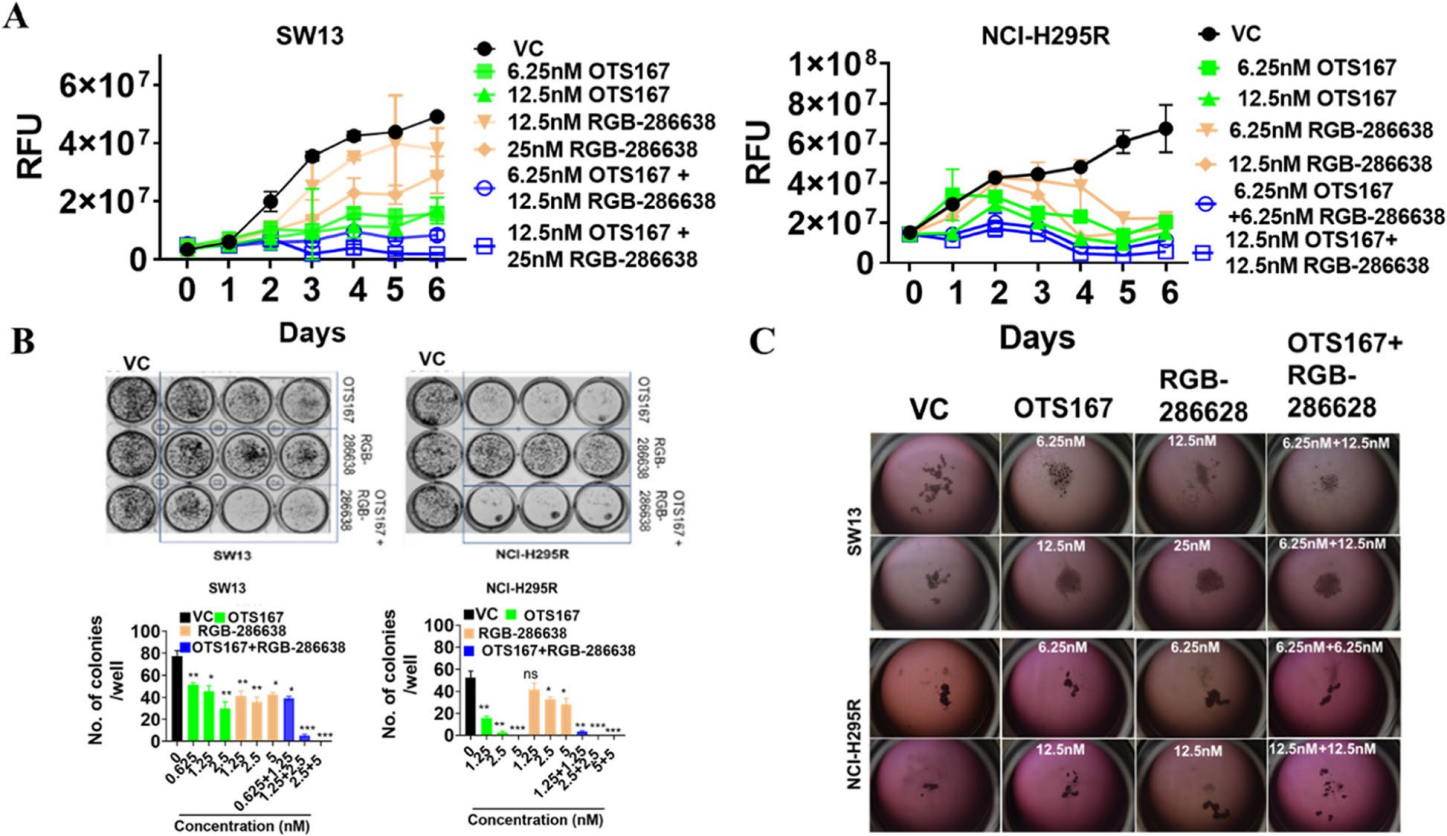
Drug combinations significantly reduce both monolayer and multilayer culture of ACC: **A** Effects of the OTS167 and RGB-286638 drugs individually or in different combinations up to six days in two different ACC cell lines, SW13 (left panel) and NCI-H295R (right panel), by cell proliferation assay. **B** Effects of OTS167 and RGB-286638 individually and in combination on colony formation in ACC cells. Graphs represent the mean colony counts of SW13 cells treated with OTS167 (0.625 nM,1.25 nM), RGB-286638 (1.25 nM, 2.5 nM), and OTS167 and RGB-286638 combined and of NCI-H295R cells treated with OTS167 (1.25 nM, 2.5 nM), RGB-286638 (1.25 nM, 2.5 nM), and OTS167 and RGB-286638 combined. Error bars are mean ± SEM. **C** Effects of OTS167 and RGB-286638 individually and in combination on multicellular aggregate formation in SW13 and NCI-H295R cells

We studied the antiproliferative effect of OTS167 and RGB-286638 alone and in combination in monolayer cell culture and MCAs. Dose-dependent antiproliferative and cytotoxic effects occurred at clinically achievable concentrations after treatment. The combination treatment resulted in a significantly higher antiproliferative effect in both cell lines compared to controls. After testing multiple-dose combinations, we found that the CI was < 1 by the Chou–Talalay method, consistent with synergistic activity (Fig. 3A and Supplementary Table 4).

The combination treatment reduced colony formation more effectively than the single-drug or vehicle groups (*P* < 0.001)(Fig. 3B).

MCAs mimic three-dimensional tumors more accurately than a monolayer culture. The combination of OTS167 and RGB-286638 was more effective than the single-drug treatment in SW13, with the disintegration of MCAs after two weeks (Fig. 3C). MCAs of NCI-H295R with combination treatment present as small and scattered foci compared to controls (Fig. 3C).

### OTS167 and RGB-286638 combination treatment induces caspase-dependent apoptosis

**Fig. 4:**
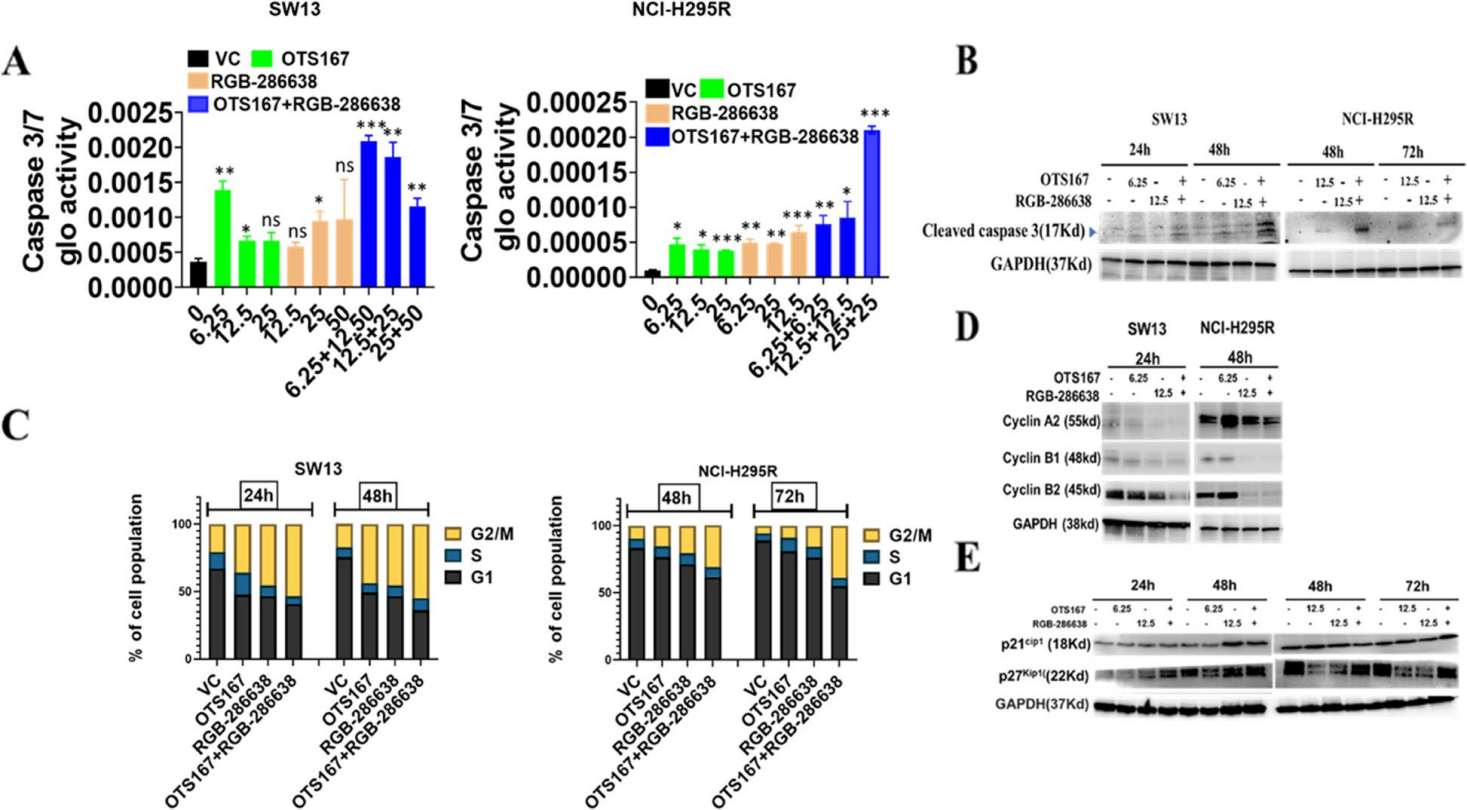
Drug combinations induce caspase-dependent apoptosis and G2/M phase and regulate cell cycle regulatory molecules: **A** Caspase-3/7 activity in SW13 and NCI-H295R cells treated with OTS167, RGB-286638, and OTS167 and RGB-286638 combined, analyzed by Caspase-Glo 3/7 assay. **B** Expression of cleaved caspase-3 in SW13 (left panel) and NCI-H295R (right panel) cells treated with OTS167, RGB-286638, and OTS167 and RGB-286638 combined, analyzed by Western blot analysis. **C** SW13 and NCI-H295R cells were treated for 24–48 h and 48–72 h, respectively. Cell cycles were analyzed with PI staining. Bar graphs represent the percentage (%) of cells in G2/M, S, and G1 phase in SW13 (left panel) and NCI-H295R (right panel) cells. **D** Expression of Cyclin A2, Cyclin B1 and Cyclin B2 in SW13 and NCI-H295R cells treated with OTS167, RGB-286638, and OTS167 and RGB-286638 combined. **E** Expression of p21^Cip1^ and p27^Kip1^ in SW13 cells (left panel) treated for 24–48 h and NCI-H295R cells (right panel) treated for 48–72 h with OTS167, RGB-286638, and OTS167 and RGB-286638 combined

We evaluated caspase-3/-7 activity, which revealed co-treatment with OTS167 and RGB-286638 in different dose combinations increased caspase-dependent apoptosis as compared to controls in both cell lines (Fig. 4A). Validation studies by Western Blot and immunofluorescence showed higher cleaved caspase-3 expression in both cell lines with combination treatment (Fig. 4B and Supplementary Fig. 4).

### OTS167 and RGB-286638 combination treatment induces G2/M cell cycle arrest

We observed an increased ratio of cells in G2/M arrest with combination treatment compared to single-drug or vehicle control (Fig. 4C).

Next, we validated the effects of combination treatment on the prognostically significant cyclins (Fig. 4D). The combination treatment downregulated protein expression of these cyclins and upregulated the expressions of p21^Cip1^ and p27^Kip1^ compared to controls in both cell lines (Fig. 4D and E).

### OTS167 and RGB-286638 combination treatment inhibits cell migration, invasion, and EMT

**Fig. 5:**
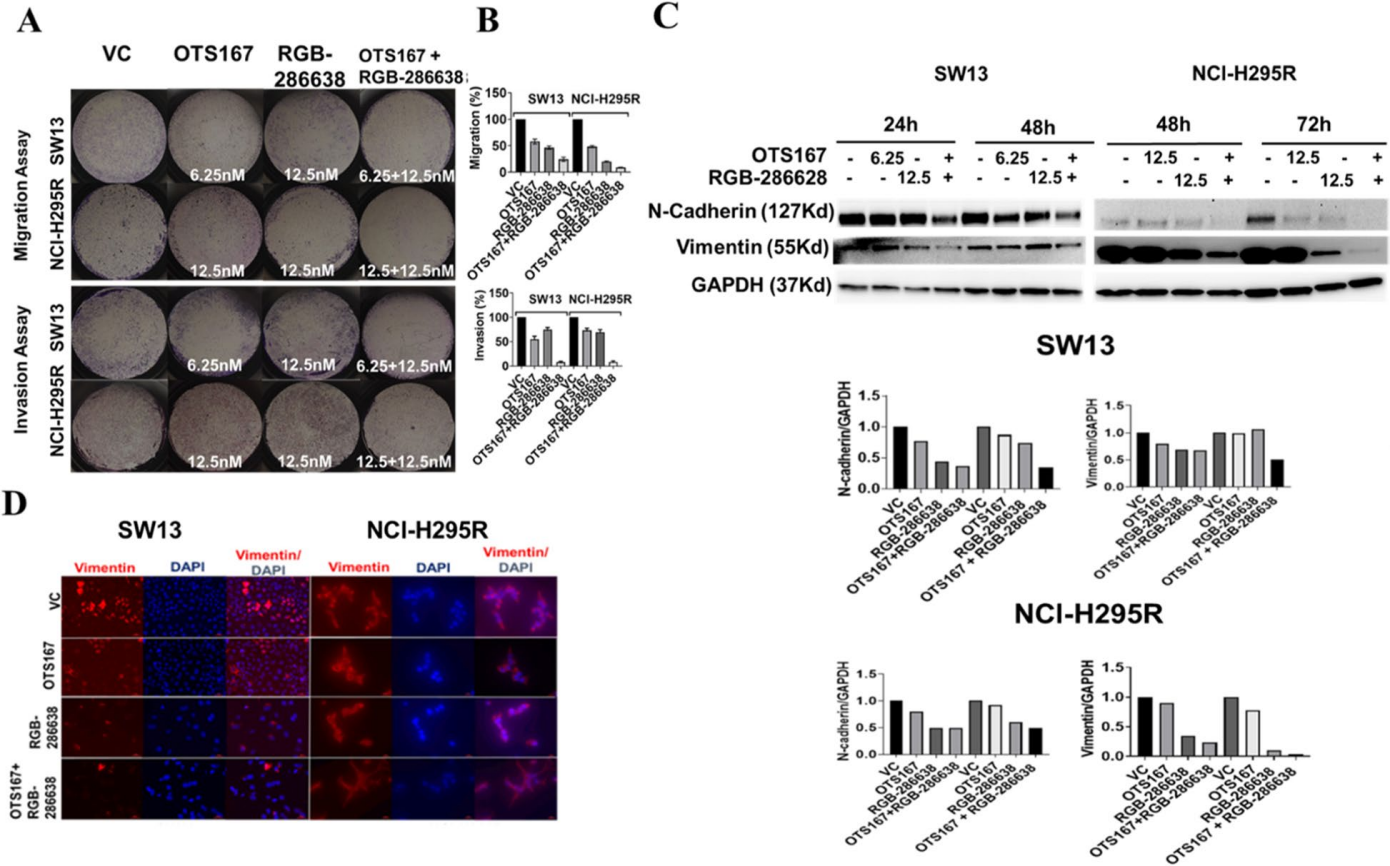
Drug combination inhibits cell invasion and migration and induces EMT markers: **A** SW13 and NCI-H295R cells were treated with OTS167, RGB-286638, and a combination of OTS167 and RGB-286638. Migration (upper two panels) and Matrigel invasion (lower two panels) assays were performed at 24 h and 48 h for SW13 and NCI-H295R cells, respectively. Images were taken at 20X magnification. Data are representative of at least three independent experiments. **B** Percentages (%) of migrated and invaded cells in respect to control in each well of SW13 and NCI-H295R cells treated with OTS167, RGB-286638, and OTS167 and RGB-286638 combined. **C** Expression of N-cadherin and vimentin in SW13 and NCI-H295R cells treated with OTS167, RGB-286638, and OTS167 and RGB-286638 combined, analyzed by Western blot analysis. **D** Expression of vimentin in SW13 cells treated for 48 h and NCI-H295R cells treated for 72 h with OTS167, RGB-286638, and OTS167 and RGB-286638 combined, analyzed by immunofluorescence analysis

ACC-related mortality is often caused by progressive metastasis as cancer cells migrate and invade the metastatic sites [20]. We found that OTS167 and RGB-286638 combination treatment decreased ACC migration and invasion compared to controls and single-drug in both cell lines (Fig. 5A and B). Next, we studied the EMT markers due to their association with cellular invasion and migration [21]. The combination treatment downregulated N-cadherin and vimentin in both ACC cell lines compared to control groups (Fig. 5C). We validated the lower vimentin expression by immunofluorescence analysis (Fig. 5D).

### OTS167 and RGB-286638 combination treatment downregulates MELK and key molecules in the β-catenin pathway

β-catenin (*CTNNB1*) overexpression is a key oncogenic signaling pathway in ACC [9]. We first evaluated the correlations between the mRNA expression of genes involved in Wnt/β-catenin signaling and the mRNA expression of *MELK* in the TCGA cohort. The mRNA expression of *FOXM1* positively and strongly correlated with *MELK* (Supplementary Table 5). Next, we confirmed that ACC overexpressed *FOXM1* in two GEO cohorts (Supplementary Fig. 5A). *FOXM1* mRNA expression analyses in TCGA revealed that overexpression was associated with statistically shorter OS, DFS, higher overall stage and T stage, and positive lymph nodes (Supplementary Fig. 5B-F).

**Fig. 6:**
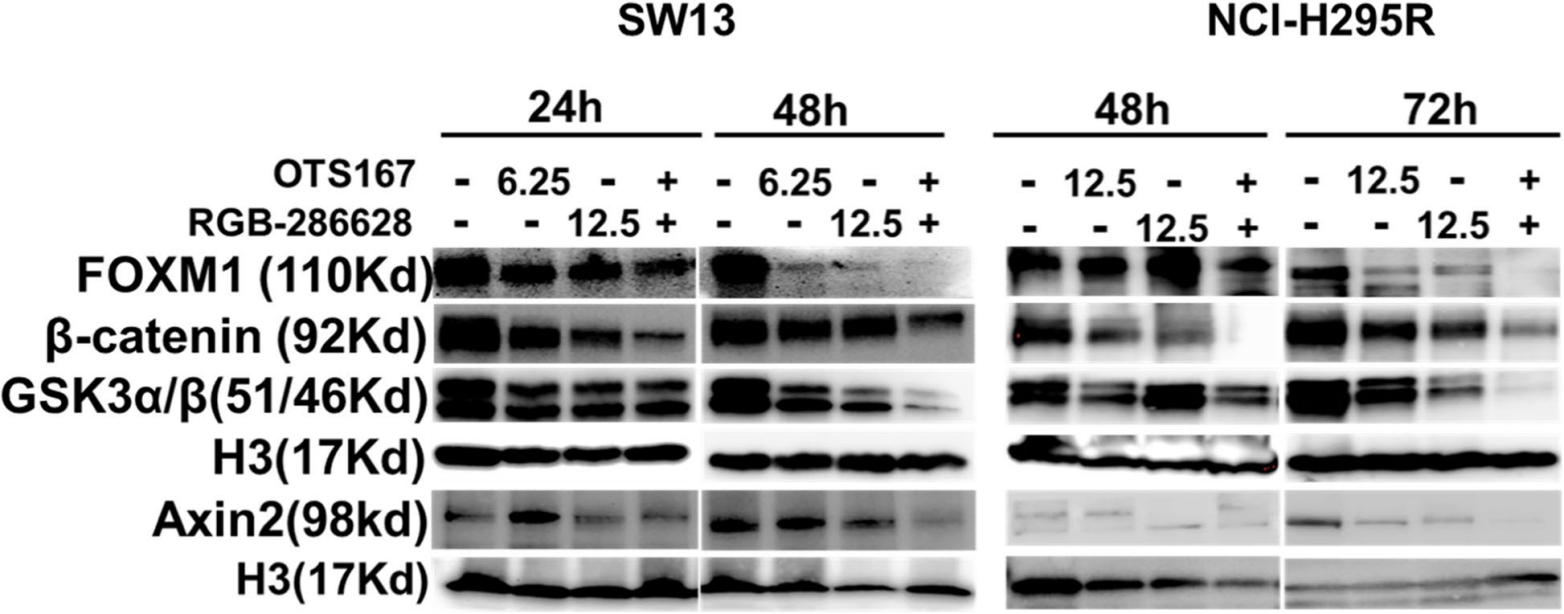
Drug combination inhibits multiple molecules in Wnt/β-catenin signaling pathway in ACC: SW13 and NCI-H295R cells were treated with OTS167, RGB-286638, and a combination of OTS167 and RGB-286638. Western blot analysis for β-catenin, AXIN2, GSK3α/β, and FOXM1 expression in SW13 cells treated for 24–48 h and NCI-H295R cells treated for 48–72 h with OTS167, RGB-286638, and OTS167 and RGB-286638 combined is shown

We then confirmed significant positive correlations in mRNA expression between *CTNNB1* and other genes that regulate the β-catenin signaling pathway (Supplementary Table 5). Compared to control groups, the combination of OTS167 and RGB-286638 downregulated FOXM1, AXIN2, GSK-3α/β, and β-catenin protein in SW13 and NCI-H295R cells (Fig. 6). We performed at least three repeated experiments with consistent results. We found that OTS167 downregulated MELK expression in a dose- and time-dependent manner (Supplementary Fig. 6A). We then treated cells with siMELK, which resulted in downregulation of FOXM1, AXIN2, GSK-3β, and β-catenin, suggesting that MELK is the upstream regulator of these molecules in the Wnt/β-catenin signaling pathway in ACC (Supplementary Fig. 6B).

### OTS167 and RGB-286638 combination treatment decreases stathmin expression and microtubule stability

**Fig. 7:**
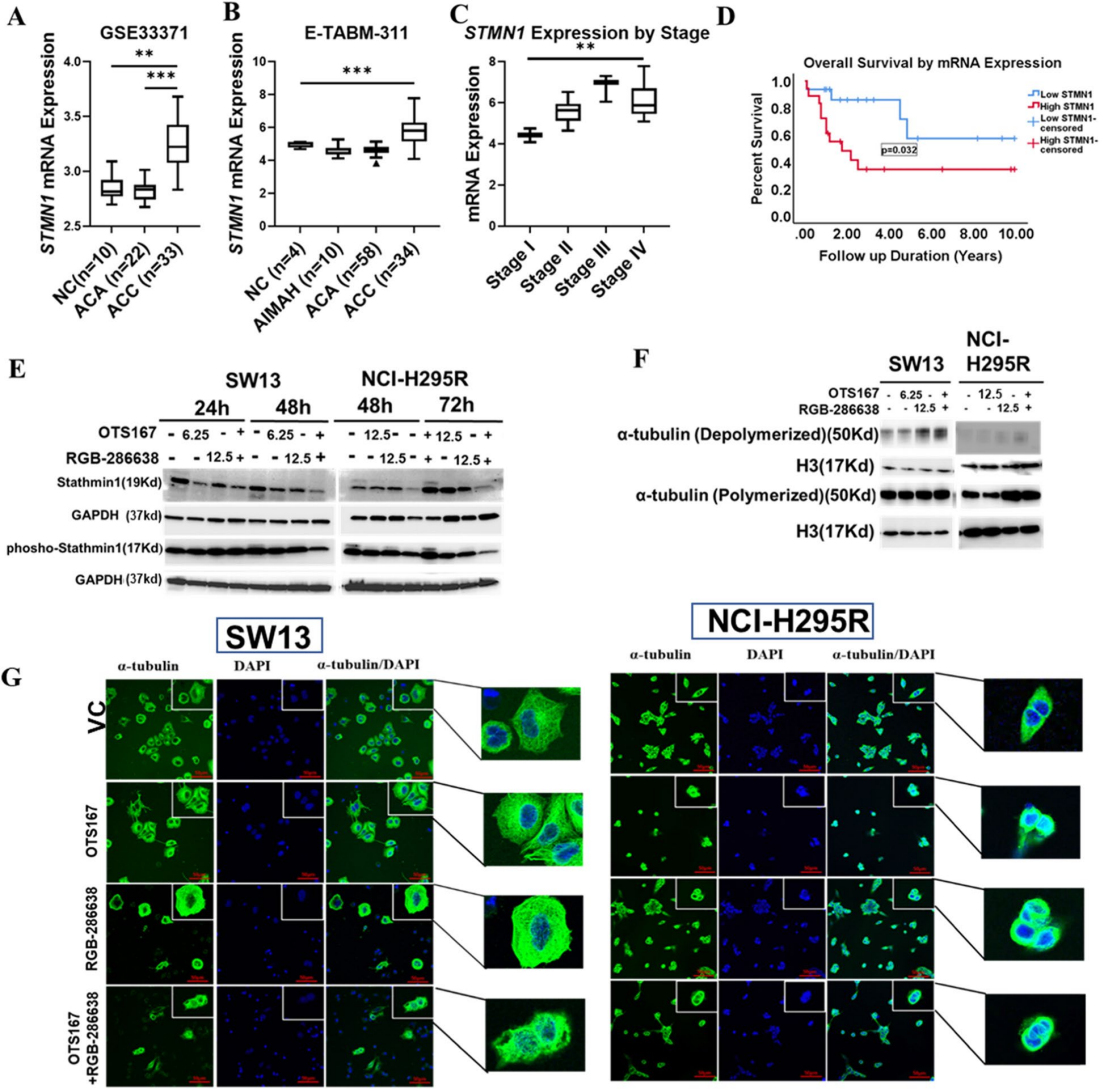
Drug combination inhibits STMN1 expression in ACC: **A** mRNA expression of *STMN1* in NC (*n* = 10), ACA (*n* = 22), and ACC (*n* = 35) in the GSE33371 data set. **B** mRNA expression of *STMN1* in NC (*n* = 4), AIMAH (*n* = 10), ACA (*n* = 58), and ACC (*n* = 34) in the E-TABM-311 cohort. **C** Differential mRNA expression of *STMN1* by tumor stages in E-TABM-311 ACC patient samples (*p* < 0.01). **D** Kaplan–Meier survival curve representing the OS in ACC patient samples from E-TABM-311 with high and low *STMN1* expression. High *STMN1* expression (red); low *STMN1* expression (blue) (*p < 0.05). **E** STMN1 expression in SW13 cells treated for 24–48 h and NCI-H295R cells treated for 48–72 h with OTS167, RGB-286638, and OTS167 and RGB-286638 combined, analyzed by Western blot analysis. **F** Expression of depolymerized and polymerized tubulin in SW13 cells treated for 24 h and NCI-H295R cells treated for 48 h with OTS167, RGB-286638, and OTS167 and RGB-286638 combined. **G** Immunofluorescence images of tubulin structure. SW13 and NCI-H295R cells treated with OTS167, RGB-286638, and OTS167 and RGB-286638 combined for 24 and 48 h respectively were labeled with α-tubulin (green), and DAPI (blue) for nuclei staining. Images were taken under confocal microscopy at 20X magnification

Stathmin (STMN1) regulates microtubule dynamics for cell cycle regulation and has strong and statistically significant correlation coefficients with *CDK1, CDK2*, and their partnering cyclins molecules (Supplementary Table 6). We found that human ACC samples overexpressed *STMN1* compared to NC and ACA in a GEO cohort (Fig. 7A). A previous study showed that *STMN1* overexpression in ACC was associated with adverse prognostic clinical features and shorter OS [22]. We validated this finding in an independent cohort using the E-TABM-311 database. *STMN1* mRNA expression is higher in ACC samples in the E-TABM-311 cohort as compared to NC, adrenocorticotropic hormone-independent macronodular adrenal hyperplasia (AIMAH), and ACA (*p* < 0.001) (Fig. 7B). In addition, *STMN1* overexpression was significantly associated with stage IV disease and shorter OS (Fig. 7C, D).

We next evaluated the effect of OTS167 and RGB-286638 on total STMN1 and p-STMN1 expression. Compared to controls, combination drug treatment decreased total and p-STMN1 expression in both ACC cell lines (Fig. 7E). STMN1 destabilizes microtubules and is negatively regulated by phosphorylation during mitosis by CDKs. STMN1 has two distinct activities: 1) reduce microtubule polymer by sequestration of tubulin and 2) direct binds to microtubule and promotes depolymerization of microtubules and preventing the microtubule assembly required for cells to build the mitotic spindle [23]. In contrast, STMN1 phosphorylation weakens STMN1-tubulin binding and increases the concentration of cytoplasmic tubulin for microtubule assembly [24]. The combination treatment increased depolymerized tubulin in ACC cells, consistent with the expected effect caused by decreased p-STMN1. In NCI-H295R cells, we also observed an upregulation of polymerized α-tubulin, possibly from decreased microtubule polymer sequestration due to downregulation of total STMN1 [25] (Fig. 7F).  Furthermore, we assessed the treatment effect on cytoskeletal structure by evaluating α-tubulin expression and pattern of microtubule distribution using immunofluorescence (Fig. 7G). While untreated SW13 and NCI-H295R cells showed normal microtubule distribution expanding throughout the cytoplasm, ACC cells treated with OTS167, RGB-286638, and the combination drugs exhibited irregular and disorganized microtubule structure and distribution (Fig. 7G). The antibody details are included in supplementary table 7.

### OTS167 and RGB-286638 combination treatment reduces tumor burden in an NCI-H295R human ACC xenograft model

**Fig. 8:**
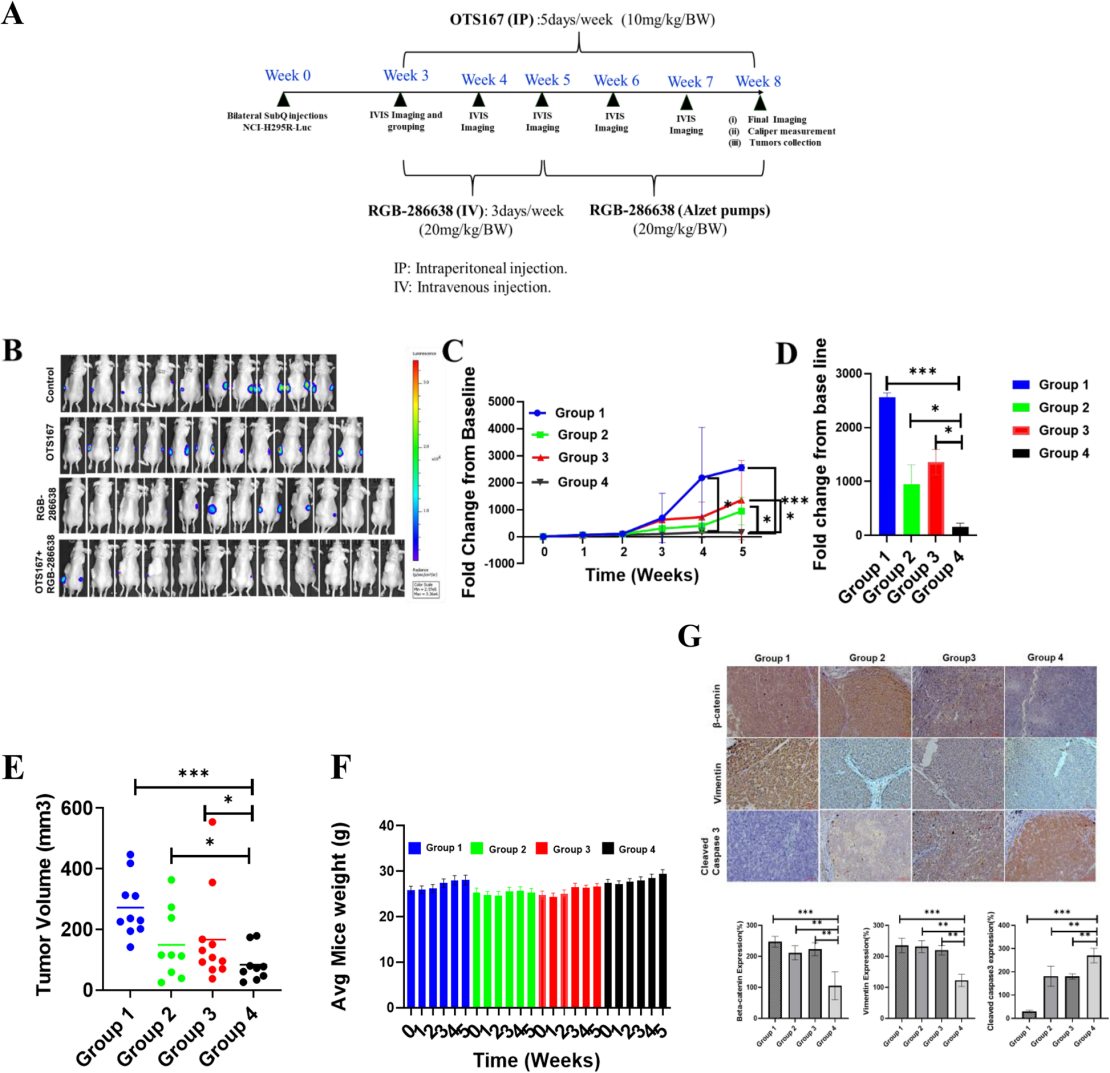
Drug combinations reduce tumor volume and luciferase signal in an NCI-H295R-Luc-cell-mediated xenograft model: **A** Schematic diagram showing treatment times, dosages, drug delivery routes and endpoint of the study**. B** Effects of OTS167, RGB-286638, and a combination of OTS167 and RGB-286638 in NCI-H295R-Luc mediated xenograft tumors in athymic nude mice. Luciferase level NCI-H295R cells were implanted in athymic nude mice. Bioluminescence images of NCI-H295R subcutaneous xenografts are shown. The upper row shows mice treated with vehicle control (Group 1); the middle two rows (Group 2 and 3) show mice treated with 10 mg/kg/BW OTS167 and 20 mg/kg/BW RGB-286638, respectively; and the bottom row (Group 4) shows mice treated with a combination of OTS167 and RGB-286638. **C** Weekly fold change of the luciferase signal of each group (Group 1, Group 2, Group 3, and Group 4). All data were analyzed by two-tailed unpaired one-way ANOVA. **D** Fold change of the luciferase signal of each group (Group 1, Group 2, Group 3, and Group 4) at the final day (week 5). **E** Caliper measurement of tumor volume at the final day (week 5) of treatment in different groups. Tumor sizes were unmeasurable due to the smaller size in one mouse from groups 1 and 3, two mice from group 2, and three mice from group 4. **F** Average weight of mice measured weekly from each group. **G** IHC study of β-catenin, vimentin, and cleaved caspase-3 from mouse tumor tissue. The bottom graph represents the percentage (%) of β-catenin, vimentin, and cleaved caspase-3 expression in Group 2 (OTS167 treatment), Group 3 (RGB-286638 treatment), and Group 4 (OTS167 and RGB-286638 combination treatment) with respect to Group 1 (vehicle treatment)

We assessed the anti-tumor efficacy of the combination therapy in vivo using a subcutaneously implanted human ACC xenograft model. We included i*n vivo* study schema in Fig. 8A. Mice treated with the combination of OTS167 and RGB-286638 had markedly lower tumor burden measured by luciferase activity and tumor volume after five weeks compared to single-drug and vehicle control groups (Fig. 8B–E). We observed no significant change in animal weight or well-being during the treatment and observation period, suggesting that there were no serious treatment-related toxicities (Fig. 8F).

ACC xenografts treated with the drug combination had lower expressions of β-catenin and vimentin with higher expression of cleaved caspase-3 by IHC as compared to control groups, which validated in vitro findings (Fig. 8G).

## Discussion

We aimed to address a lack of effective systemic treatments for patients with advanced ACC. We used a drug repurposing approach by performing a qHTS campaign using a comprehensive library comprising 4,991 experimental and approved drugs against two ACC cell lines. To facilitate clinical trial development, we prioritized drugs with known toxicity profiles, pharmacokinetics, and pharmacodynamics in humans with efficacy against ACC cells at clinically achievable concentrations. Because ACC is often refractory to systemic therapy, we aimed to identify synergistic drug combinations and study synergistic mechanisms to improve patient selection, treatment efficacy, and outcome. We identified OTS167 and RGB-286638 from the combination matrix drug screening as they demonstrated potent synergy. OTS167 and RGB-286638 each had higher efficacy against ACC cells than the current chemotherapy for ACC. OTS167 (OncoTherapy Science, Japan) is an orally available potent small-molecule inhibitor of MELK. It is currently in Phase I clinical trials in patients with breast cancers and leukemia. RGB-286638 (Aggenix AG, Germany) is a multi-CDK small molecule inhibitor with additional activity against tyrosine kinases and serine/threonine kinases and was well tolerated with prolonged disease stabilization in Phase I clinical trials in patients with solid tumors [17]. We chose the combination of MELK (OTS167) and CDK (RGB-286638) inhibitors because 1) ACC overexpressed MELK, CDK1, CDK2, and partnering cyclins, and 2) these therapeutic targets of OTS167 and RGB-286638 were associated with advanced ACC and independently associated with shorter OS and DFS in several databases, suggesting their role in ACC progression [10]. Compared to our previous study that manually identified the effective combination based on mechanisms of action [26], we utilized high-throughput technology with pairwise combination matrix screening to identify the synergy regardless of their mechanisms. In this study, we showed that the combination treatment was more effective against in vitro and in vivo ACC cell proliferation than single-drug treatments. We confirmed that the drug combination induced G2/M cell cycle arrest and caspase-dependent apoptosis. Because RGB-286638 inhibits the enzymatic activity of CDKs, we assessed the expression of other cell cycle regulators as these can be clinically used to aid patient selection and assess treatment response. CDK1 is the key determinant of mitotic progression, while CDK2 plays a pivotal role in DNA replication. Encoded by the *cdc2* gene, CDK1 is activated by and complexes with cyclins A2, B1, and B2 to phosphorylate key proteins leading to mitosis [27, 28]. CDK2 and A- and E-type cyclins form active kinase complexes to phosphorylate proteins involved in DNA replication, which are inactivated by the cell cycle checkpoints p21^Cip1^ and p27^Kip1^ [29]. Dysregulation of CDK1, CDK2, and their partner cyclins has been observed in multiple solid cancers with aggressive behavior [30–33]. Similar to the overexpression of *CDK1* and *CDK2* in ACC, we confirmed associations between *CCNA2*, *CCNB1*, *CCNB2*, and *CCNE2* mRNA overexpression and adverse clinical features. We demonstrated that the drug combination effectively decreased cyclin expression, induced p21^Cip1^ and p27^Kip1^, and caused G2/M cell cycle arrest, suggesting that the combination of OTS167 and RGB-286638 preferentially targets the molecules associated with ACC aggressiveness.

One of the key mechanisms leading to cancer metastasis is the EMT-related cellular invasion [34]. The EMT has also been implicated in the generation of cancer stem cells and in chemo- and radio-resistance [35]. The molecular characteristics of EMT include the downregulation of epithelial markers such as E-cadherin and the upregulation of mesenchymal markers such as vimentin and N-cadherin. The combination of OTS167 and RGB-286638 effectively decreased ACC cell migration and invasion and reduced vimentin and N-cadherin expressions, possibly via the downregulation of the Wnt/β-catenin signaling pathway [36].

MELK has been shown to activate mitotic regulatory genes by activating *FOXM1* in other cancers [37]. We analyzed TCGA data and confirmed a positive linear correlation between *MELK* and *FOXM1* mRNA expressions, suggesting the *MELK*-dependent activation of *FOXM1* also occurs in ACC. We validated this hypothesis by showing that *FOXM1* overexpression is associated with adverse clinical features in ACC. Additionally, 41% of ACC samples in TCGA harbored alterations of the Wnt/β-catenin pathway. Preclinical studies of glioma cells showed that FOXM1 directly interacts with β-catenin and promotes nuclear translocation [38]. Downregulation of the β-catenin pathway using a doxycycline-inducible shRNA plasmid inhibited ACC cell proliferation, induced apoptosis, and inhibited in vivo ACC tumor growth [39]. Although these findings suggested that the Wnt/β-catenin pathway is a promising therapeutic target in ACC, current therapies do not address this key oncogenic driver. The novel combination of OTS167 and RGB-286638 downregulated multiple clinically relevant key molecules in the Wnt/β-catenin pathway, such as FOXM1, Axin2, GSK3-β, and nuclear and cytoplasmic β-catenin.

In addition to the role of FOXM1 in cell cycle progression, a recent study showed that FOXM1 is a master regulator of hepatocellular carcinoma metastasis by inducing EMT and increasing cell migration by transcriptionally activating *STMN1* [40]. The altered cytoskeleton caused by the microtubule destabilizing activity of STMN1 increases cell motility and plays a critical role in cell migration, invasion [41], and proliferation. STMN1 also regulates cell cycle progression by controlling tubulin polymerization and depolymerization, and its inhibition results in the G2/M cell cycle arrest [42]. Previous work showed that *STMN1* overexpression in ACC promoted a more aggressive in vitro phenotype and was associated with shorter OS in the TCGA cohort [22]. Once we confirmed the positive correlations between *STMN1* mRNA expression to *FOXM1*, *MELK*, *CDK1,* and *CDK2*, we validated the *STMN1* overexpression in advanced ACC and the association with shorter disease-specific survival from the E-TABM-311 cohort. Our work demonstrated that the combination of OTS167 and RGB-286638 decreased total and p-STMN1, resulting in increased depolymerized α-tubulin in ACC cells. Because STMN1 sequestered tubulin polymer, the increased polymerized microtubule in ACC treated with OTS167 and RGB-286638 was consistent with a previous report [25]. Our data suggest that this drug combination inhibited MELK and FOXM1, resulting in the downregulation of total and p-STMN1, affecting microtubule dynamics. We confirmed that the treatments with OTS167 and RGB-286638 caused disorganized microtubule and irregular cytoskeletal distribution in ACC cells. Such effects on the ACC cytoskeleton can lead to decreased motility, invasion, and mitotic spindle formation and resulting in G2/M cell cycle arrest.

The increased caspase-dependent apoptosis in ACC xenografts treated with OTS167 and RGB-286638 validated the in vitro synergy of this combination. We also confirmed the loss of cytoplasmic and nuclear expression of β-catenin in human ACC xenografts. Thus, we believe that the combination of OTS167 and RGB-286638 addresses the key molecular characteristics involved in ACC tumorigenesis and metastasis. This regimen should be studied in patients with advanced ACC, especially tumors with higher expression of Ki67, MELK, CDK1, CDK2, their partnering cyclins, FOXM1, STMN1, and CTNNB1. In addition to in vivo validation of anti-tumor efficacy of the combination treatment in human ACC xenografts, we found that mice tolerated the combination treatment well with no signs of systemic toxicities until the endpoint was met at week 5. Nevertheless, a phase Ib dose-escalation of OTS167 and RGB-286638 in patients with advanced ACC with careful monitoring of major organ functions is still required to assess safety and tolerability and to identify the recommended phase II dose.

## Conclusions

In summary, we addressed an urgent need to identify effective systemic treatment in patients with advanced ACC by identifying a potent antineoplastic drug combination through qHTS in ACC in vitro. We validated the in vitro and in vivo efficacy of OTS167 and RGB-286638 and discovered that this drug combination effectively targeted MELK, FOXM1, STMN1, and multiple clinically significant molecules in the Wnt/β-catenin signaling pathway, resulting in an antiproliferative and cytotoxic effect, inhibition of cell migration and invasion, and clonogenicity. A clinical trial to assess the safety and efficacy of these compounds is warranted.

## Supplementary Information

**Additional file 1.** Supplementary methods.

**Additional file 2: Supplementary Figure 1.** CDKs and cyclin molecules are overexpressed in ACC: **A** mRNA expression of *CDK1* and *CDK2 *(NC vs. ACC) from the publicly available data set GSE90713 (NC = 5, ACC = 57) (*p* < 0.05). **B** mRNA expression of *CCNA2*, **C** mRNA expression of *CCNB1*, **D** mRNA expression of *CCNB2*, and E mRNA expression of *CCNE2*, from the GSE33371, GSE12638, and GSE90713 data sets. **Supplementary Figure 2.** Cyclin molecule overexpression correlates with poor prognosis in ACC: **A** Kaplan-Meier survival curve representing the OS in the TCGA ACC cohort with low and high expression of *CCNA2*, *CCNB1*, and *CCNB2*. **B** Kaplan-Meier survival curve representing the overall DFS in the TCGA ACC cohort with low and high expression of *CCNA2*, *CCNB1*, and *CCNB2*. **C, D** Differential mRNA expression of *CCNA2*,* CCNB1, *and *CCNB2* by over all stages and T stage in the TCGA ACC cohort. All data were analyzed by two-tailed unpaired Student’s t-test or one-way ANOVA. **Supplementary Figure 3. **Cyclin E2 is associated with poor prognosis of patients with ACC: **A, B** Kaplan-Meier survival curve representing the overall and DFS in the TCGA ACC cohort with high and low *CCNE2*expression. High *CCNE2* expression (red); low *CCNE2* expression (blue) (*p < 0.01). **C** *CCNE2* expression by over all stage and T stage in the TCGA ACC cohort (*p < 0.05). **Supplementary Figure 4. **Cleaved Caspase 3 expression in SW13 and NCI-H295R cells treated with OTS167, RGB-286638, and OTS167 and RGB-286638 combined for 24 and 48 hours respectively. Cells were stain with cleaved caspase 3 conjugated with Alexa Fluor® 555 Conjugate (Red). Actin filaments and nuclei were labeled with Alexa Fluor 488 phalloidin (green) and DAPI respectively. The images were taken under confocal microscopsy (Zeiss) at 63X. **Supplementary Figure 5.** FOXM1 is overexpressed in advanced tumor stage and correlated with poor prognosis of ACC patient: **A** mRNA expression of *FOXM1 *in the publicly available data sets GSE33371 (NC vs. ACC, ACA vs. ACC) (*p* < 0.001) and GSE12368 (NC vs. ACC, ACA vs. ACC) (*p* < 0.05). **B, C** Kaplan-Meier survival curve and DFS curve of patients with ACC, stratified by *FOXM1* expression level. **D, E, F** Differential expression of *FOXM1* mRNA by stage, tumor grade status, and lymph node status. **Supplementary Figure 6.** MELK inhibition affects β-catenin pathway in ACC cells: **A** SW13 and NCI-H295R cells were treated with OTS167. Expression of MELK in SW13 and NCI-H295R cells treated with OTS167 for 24–72 hours. **B** Expression of β-catenin, FOXM1, GSK-3β, and Axin 2 in siMELK and siNegative transfected cells.

**Additionalfile 3: Supplementary Table 1.** List of 30 selected drugs for combination drug screening in ACC cell lines. **Supplementary Table 2.** Correlations between mRNA expression of CDKs and cyclin molecules with MKI67 in human ACC from the TCGA cohort **Supplementary Table 3. **Correlations between mRNA expression of CDKs and cyclin molecules with MELK in human ACC samples from the TCGA cohort. **Supplementary Table 4. **OTS167 and RGB-286638 combination shows synergistic activity in ACC cells. The combination index (CI) was calculated using the Chou–Talalay method. The CI is determined by the following range: CI < 1, synergistic; CI = 1, additive; CI > 1, antagonistic. **Supplementary Table 5. **Correlations between mRNA expression of MELK and β-catenin regulatory molecules in human ACC samples from the TCGA cohort **Supplementary Table 6. **Correlations between mRNA expression of CDKs and cyclin molecules with STMN1 in human ACC samples from the TCGA cohort **Supplementary Table 7. **List of Antibodies.

## Data Availability

The datasets supporting the conclusions of this article are included within the article. Datasets are described in the material and method section.

## Abbreviations

ACC: Adrenocortical Carcinoma
MELK: Maternal embryonic leucine zipper kinase
CDK: Cyclin-dependent kinase
FOXM1: Forkhead box protein M1
GEO: Gene Expression Omnibus
TCGA: The Cancer Genome Atlas
GSK3-β: Glycogen synthase kinase 3 beta
qHTS: Quantitative high-throughput drug screening
EMT: Epithelial to mesenchymal transition
OS: Overall survival
ENSAT: European Network for the Study of Adrenal Tumors
ACA: Adrenocortical adenoma
NC: Normal cortex
E-TABM-311: European Bioinformatics Institute
IHC: Immunohistochemistry
PBS: Phosphate buffer saline

## References

[1] Wooten MD, King DK (1993). Adrenal cortical carcinoma. Epidemiology and treatment with mitotane and a review of the literature. Cancer..

[2] Kebebew E, Reiff E, Duh QY, Clark OH, McMillan A (2006). Extent of disease at presentation and outcome for adrenocortical carcinoma: have we made progress?. World J Surg.

[3] Kerkhofs TM, Verhoeven RH, Van der Zwan JM, Dieleman J, Kerstens MN, Links TP (2013). Adrenocortical carcinoma: a population-based study on incidence and survival in the Netherlands since 1993. Eur J Cancer.

[4] Fassnacht M, Dekkers OM, Else T, Baudin E, Berruti A, de Krijger R (2018). European Society of Endocrinology Clinical Practice Guidelines on the management of adrenocortical carcinoma in adults, in collaboration with the European Network for the Study of Adrenal Tumors. Eur J Endocrinol.

[5] Fassnacht M, Kroiss M, Allolio B (2013). Update in adrenocortical carcinoma. J Clin Endocrinol Metab.

[6] Wang S, Chen SS, Gao WC, Bai L, Luo L, Zheng XG (2017). Prognostic Factors of Adrenocortical Carcinoma: An Analysis of the Surveillance Epidemiology and End Results (SEER) Database. Asian Pac J Cancer Prev.

[7] Fassnacht M, Terzolo M, Allolio B, Baudin E, Haak H, Berruti A (2012). Combination chemotherapy in advanced adrenocortical carcinoma. N Engl J Med.

[8] Assie G, Letouze E, Fassnacht M, Jouinot A, Luscap W, Barreau O (2014). Integrated genomic characterization of adrenocortical carcinoma. Nat Genet.

[9] Zheng S, Cherniack AD, Dewal N, Moffitt RA, Danilova L, Murray BA (2016). Comprehensive Pan-Genomic Characterization of Adrenocortical Carcinoma. Cancer Cell.

[10] Nilubol N, Boufraqech M, Zhang L, Gaskins K, Shen M, Zhang YQ (2018). Synergistic combination of flavopiridol and carfilzomib targets commonly dysregulated pathways in adrenocortical carcinoma and has biomarkers of response. Oncotarget.

[11] Martinez NJ, Rai G, Yasgar A, Lea WA, Sun H, Wang Y (2016). A High-Throughput Screen Identifies 2,9-Diazaspiro[5.5]Undecanes as Inducers of the Endoplasmic Reticulum Stress Response with Cytotoxic Activity in 3D Glioma Cell Models. PLoS One.

[12] Mathews Griner LA, Guha R, Shinn P, Young RM, Keller JM, Liu D (2014). High-throughput combinatorial screening identifies drugs that cooperate with ibrutinib to kill activated B-cell-like diffuse large B-cell lymphoma cells. Proc Natl Acad Sci U S A.

[13] Edgar R, Domrachev M, Lash AE (2002). Gene Expression Omnibus: NCBI gene expression and hybridization array data repository. Nucleic Acids Res.

[14] Chou TC (2010). Drug combination studies and their synergy quantification using the Chou-Talalay method. Cancer Res.

[15] Ravi M, Paramesh V, Kaviya SR, Anuradha E, Solomon FD (2015). 3D cell culture systems: advantages and applications. J Cell Physiol.

[16] Maes A, Maes K, Vlummens P, De Raeve H, Devin J, Szablewski V (2019). Maternal embryonic leucine zipper kinase is a novel target for diffuse large B cell lymphoma and mantle cell lymphoma. Blood Cancer J.

[17] Cirstea D, Hideshima T, Santo L, Eda H, Mishima Y, Nemani N (2013). Small-molecule multi-targeted kinase inhibitor RGB-286638 triggers P53-dependent and -independent anti-multiple myeloma activity through inhibition of transcriptional CDKs. Leukemia.

[18] Beuschlein F, Weigel J, Saeger W, Kroiss M, Wild V, Daffara F (2015). Major prognostic role of Ki67 in localized adrenocortical carcinoma after complete resection. J Clin Endocrinol Metab.

[19] Wang Y, Lee YM, Baitsch L, Huang A, Xiang Y, Tong H (2014). MELK is an oncogenic kinase essential for mitotic progression in basal-like breast cancer cells. Elife.

[20] Jiang WG, Sanders AJ, Katoh M, Ungefroren H, Gieseler F, Prince M (2015). Tissue invasion and metastasis: Molecular, biological and clinical perspectives. Semin Cancer Biol.

[21] Bulzico D, Faria PAS, Maia CB, de Paula MP, Torres DC, Ferreira GM (2017). Is there a role for epithelial-mesenchymal transition in adrenocortical tumors?. Endocrine.

[22] Aronova A, Min IM, Crowley MJP, Panjwani SJ, Finnerty BM, Scognamiglio T (2018). STMN1 is Overexpressed in Adrenocortical Carcinoma and Promotes a More Aggressive Phenotype In Vitro. Ann Surg Oncol.

[23] Howell B, Larsson N, Gullberg M, Cassimeris L (1999). Dissociation of the tubulin-sequestering and microtubule catastrophe-promoting activities of oncoprotein 18/stathmin. Mol Biol Cell.

[24] Amayed P, Pantaloni D, Carlier MF (2002). The effect of stathmin phosphorylation on microtubule assembly depends on tubulin critical concentration. J Biol Chem.

[25] Howell B, Deacon H, Cassimeris L (1999). Decreasing oncoprotein 18/stathmin levels reduces microtubule catastrophes and increases microtubule polymer in vivo. J Cell Sci.

[26] Mueller-Klieser W (1987). Multicellular spheroids. A review on cellular aggregates in cancer research. J Cancer Res Clin Oncol.

[27] Bellanger S, de Gramont A, Sobczak-Thepot J (2007). Cyclin B2 suppresses mitotic failure and DNA re-replication in human somatic cells knocked down for both cyclins B1 and B2. Oncogene.

[28] Okamoto K, Sagata N (2007). Mechanism for inactivation of the mitotic inhibitory kinase Wee1 at M phase. Proc Natl Acad Sci U S A.

[29] Asghar U, Witkiewicz AK, Turner NC, Knudsen ES (2015). The history and future of targeting cyclin-dependent kinases in cancer therapy. Nat Rev Drug Discov.

[30] Scaltriti M, Eichhorn PJ, Cortes J, Prudkin L, Aura C, Jimenez J (2011). Cyclin E amplification/overexpression is a mechanism of trastuzumab resistance in HER2+ breast cancer patients. Proc Natl Acad Sci U S A.

[31] Karst AM, Jones PM, Vena N, Ligon AH, Liu JF, Hirsch MS (2014). Cyclin E1 deregulation occurs early in secretory cell transformation to promote formation of fallopian tube-derived high-grade serous ovarian cancers. Cancer Res.

[32] Kuhn E, Bahadirli-Talbott A, Shih IM (2014). Frequent CCNE1 amplification in endometrial intraepithelial carcinoma and uterine serous carcinoma. Mod Pathol.

[33] Li M, He F, Zhang Z, Xiang Z, Hu D (2020). CDK1 serves as a potential prognostic biomarker and target for lung cancer. J Int Med Res.

[34] Son H, Moon A (2010). Epithelial-mesenchymal Transition and Cell Invasion. Toxicol Res.

[35] Zheng HC (2017). The molecular mechanisms of chemoresistance in cancers. Oncotarget.

[36] Salomon A, Keramidas M, Maisin C, Thomas M (2015). Loss of beta-catenin in adrenocortical cancer cells causes growth inhibition and reversal of epithelial-to-mesenchymal transition. Oncotarget.

[37] Joshi K, Banasavadi-Siddegowda Y, Mo X, Kim SH, Mao P, Kig C (2013). MELK-dependent FOXM1 phosphorylation is essential for proliferation of glioma stem cells. Stem Cells.

[38] Zhang N, Wei P, Gong A, Chiu WT, Lee HT, Colman H (2011). FoxM1 promotes beta-catenin nuclear localization and controls Wnt target-gene expression and glioma tumorigenesis. Cancer Cell.

[39] Gaujoux S, Hantel C, Launay P, Bonnet S, Perlemoine K, Lefevre L (2013). Silencing mutated beta-catenin inhibits cell proliferation and stimulates apoptosis in the adrenocortical cancer cell line H295R. PLoS ONE.

[40] Liu J, Li J, Wang K, Liu H, Sun J, Zhao X (2021). Aberrantly high activation of a FoxM1-STMN1 axis contributes to progression and tumorigenesis in FoxM1-driven cancers. Signal Transduct Target Ther.

[41] Ni PZ, He JZ, Wu ZY, Ji X, Chen LQ, Xu XE (2017). Overexpression of Stathmin 1 correlates with poor prognosis and promotes cell migration and proliferation in oesophageal squamous cell carcinoma. Oncol Rep.

[42] Rubin CI, Atweh GF (2004). The role of stathmin in the regulation of the cell cycle. J Cell Biochem.

