# Supplementary material for: Preclinical assessment of synergistic efficacy of MELK and CDK inhibitors in adrenocortical cancer
Source: J Exp Clin Cancer Res. 2022 Sep 23;41:282. doi: 10.1186/s13046-022-02464-5 (PMC9502945; doi:10.1186/s13046-022-02464-5)
Supplement: Supplementary file 1 — Additional file 1. Supplementary methods. [file 13046_2022_2464_MOESM1_ESM.doc]

**Supplementary methods**

**Quantitative high-throughput screening**

SW13 and NCI-H295R cells were harvested from a T175 flask and resuspended in ITS-supplemented DMEM serum-free medium at a concentration of 300 000 cells/mL. Then 5 µL of resuspended cells were dispensed into each well of white, solid-bottom, 1536-well tissue-culture-treated plates using a multidrop combi dispenser. After overnight culture at 37 °C with 5% CO_2_, 23 nL of compounds at eight selected concentrations from the NPC/MIPE or positive control (10 mM stock of bortezomib) in DMSO was transferred to each well of the assay plate using a pin tool (Kalypsys, San Diego, CA, USA). The final concentration of the compounds in the 5 µL assay volume ranged from 0.5 nM to 46 µM. The plates were further incubated at 37 °C and 5% CO_2_ for 48 hours. Then 4 µL of CellTilter-Glo® luminescent substrate mix (Promega, Madison, WI, USA) was added to each well. The plate was incubated at room temperature for 20 minutes. A ViewLux plate reader (PerkinElmer, Waltham, MA, USA) with a clear filter was used to measure the number of metabolically competent cells.

To determine compound activity in the qHTS assay, the titration-response data for each sample was plotted and modeled by a four-parameter logistic fit yielding IC50 and efficacy (maximal response) values. Raw plate reads for each titration point were first normalized relative to positive control (doxorubicin hydrochloride, 100% inhibition) and DMSO-only wells (basal, 0%).

**Combination matrix screening**

A combination matrix screening was performed with a subset of active compounds identified by qHTS in SW13 and NCI-H295R cells. Plating of compounds in matrix format using acoustic droplet ejection and numerical characterization of synergy, additivity, and/or antagonism were conducted as described previously (1, 2). For combination matrix screening, the compounds were plated as a 10 × 10 dose-response combination matrix. Concentration ranges were selected from single-agent dose-response curves generated from the qHTS. Compounds were acoustically dispensed (10 nL/well) using an ATS-100 (EDC Biosystems) onto 1,536-well, white, solid-bottom, TC-treated plates. NCI-H295R cells were subsequently added to the plates (1 000 cells/well in 5 µL) and incubated for 72 hours at 37 °C with 5% CO_2_ under 85% humidity. Cell viability was determined by the addition of 2.5 μL of CellTiter-Glo into each well. After 15 minutes of incubation at room temperature, each sample’s luminescence intensity was measured using a ViewLux reader. DMSO (20 nL) and bortezomib (20 nL at 2.3 mM) were used as negative and positive controls, respectively. Viability resulted from single-agent or combination was normalized to the controls. The synergies were characterized using the Bliss independence model and summarized using the DBSumNeg metric. In brief, the Bliss independence model expected no mechanistic interaction between two tested compounds. Therefore, the viability from an additive dose combination (Cadditive) is the multiplication of factional viability upon treatment of compound X and Y individually: Cadditive = X × Y (0 ≤ X ≤ 1, 0 ≤ Y ≤ 1). The difference between measured viability and expected viability from an additive dose combination (Cmeasured - Cadditive), so called deltaBliss (DB), describes the additivity (DB = 0), synergy (DB < 0), or antagonism (DB > 0). To evaluate the overall synergy from all 81 dose combinations we tested in a 10 × 10 block, we calculated DBSumNeg as the sum of all negative DB.

**Immunohistochemistry**

The immunohistochemistry slides were gradually deparaffinized and rehydrated according to standard protocol (3). Next, the epitope retrieval was performed with citrate buffer in pressurized steam at 120 °C for 10 minutes. The endogenous peroxidase activity was blocked with 6% hydrogen peroxide (cat. #H325-30GAL, Fisher Scientific, MA, USA). The sections from human tissue were incubated with anti-MELK antibodies and Ki67 antibodies, and Mice tumor tissues were incubated with anti-β catenin, anti-vimentin, and anti-cleaved caspase-3 antibodies overnight at 4 °C, followed by an incubation with biotinylated secondary antibodies for one hour at room temperature (**Supplementary Table 7**). The slides were developed in DAB (EnVision + Kit system HRP [DAB] and then counterstained with hematoxylin. The slides were scanned at 20× magnification using a ScanScope XT digital slide scanner (Aperio Technologies, Leica) to create whole-slide image data files at a resolution of 0.5 mm/pixel, which were viewed using the ImageScope software (Aperio Technologies).

**Cellular proliferation assay**

In brief, SW13 (3 × 10^3^) and NCI-H295R (6 × 10^3^) cells were plated in 96-well black plates with a clear bottom (cat. #353219, Costar®, Corning, NY, USA). After 24 hours (day 0), the culture medium containing drugs OTS167, RGB-286638, and the combination of OTS167 and RGB-286638 at different concentrations was added to each well. The medium with the drugs or vehicle was replaced every 48 hours. Cell proliferation plates were collected for up to six days. The fluorescence intensity was determined using a fluorescence microplate reader (Molecular Devices, Sunnyvale, CA, USA) at 485 nm/538 nm.

We used the automated computerized algorithm (Chou–Talalay method) to assess whether the combination of OTS167 and RGB-286638 had synergistic efficacy. The efficacy, indicated by the combination index (CI) of the combination treatment at various doses, was compared to that of the cells treated with a single drug. CI < 1 indicated a synergistic effect; CI = 1 indicated an additive effect; and CI > 1, indicated an antagonistic effect.

### Three-dimensional multicellular aggregates (MCAs)

ACC cells were incubated at 37 °C in 5% CO_2_ for two weeks in ultra-low cluster 24-well plates. The medium was changed twice weekly. At the beginning of the third week, the cells were treated with various concentrations of single drugs, combination drugs, and vehicle control. SW13 and NCI-H295R MCAs were continuously treated for two weeks. Media containing respective drugs and vehicle control were changed twice weekly. MCAs were photographed with a Nikon D5100 (Nikon, Inc., Melville, NY, USA) under an approximately 20× magnification (Olympus SZX9 microscope with DF PLAPO 1X-2 lens, Olympus America, Inc., Center Valley, PA, USA).

**Caspase-3/-7 activation assay**

In brief, SW13 (6 × 10^3^) and NCI-H295R (6 × 10^3^) cells were cultured in 96-well white-walled clear-bottom plates (Lonza, Allendale, NJ, USA). SW13 and NCI-H295R cells were treated for 24 and 48 hours, respectively, with different concentrations of OTS167, RGB-286638, and the combination of OTS167 and RGB-286638.

After 24 hours and 48 hours of treatment, 100 µL of the Caspase-Glo 3/7 reagent (100 μL/well) was added into the 96-well white-walled clear-bottom plate. The plates were incubated in the dark for 60 minutes at room temperature on a plate shaker. Luminescence was measured in a SpectraMax® i3x plate reader (Molecular Devices, Sunnyvale, CA, USA). All treatments were performed in a triplicate manner.

**Western blot analysis**

An equal amount of proteins was resolved by electrophoresis on 4–15% or 8–20% gradient gels and transferred to PVDF membrane. We blocked the nonspecific binding to the membrane with 5% BSA in TBS-Tween buffer, then incubated the membrane at 4 °C overnight with specific primary antibodies (p21^Cip1^, p27^Kip1^, cyclin A2, cyclin B1, cyclin E2, fibronectin, vimentin, N-cadherin, β-catenin, Axin2, GSK3-α/β, FOXM1, Stathmin1, GAPDH, H3).

The membranes were incubated with horseradish-peroxidase-conjugated IgG secondary antibodies. Protein bands were analyzed using an enhanced chemiluminescence (ECL) reagent (Pierce, Thermo Fisher Scientific, Waltham, MA, USA). The quantification of the different protein levels was performed in Bio-Rad software according to the manufacturer’s instructions.

**Cellular migration and invasion assay**

SW13 and NCI-H295R cells were plated in six-well plates in a triplicate manner and treated with varying concentrations of OTS167, RGB-286638, the combination of OTS167 and RGB-286638, and vehicle control for 24 hours and 48 hours, respectively. Cells were trypsinized and plated in transwell chambers at a density of 1 × 10^5^ cells/0.5 mL medium without FBS. The bottom chambers were filled with DMEM supplemented with 10% FBS as a chemoattractant. The SW13 and NCI-H295R cells were allowed to migrate for 24 and 48 hours, respectively. The membranes of the chambers were fixed and stained with Diff-Quik (cat. #65044-93, Dade Behring, Newark, NJ, USA). Cells were imaged with approximately 20× magnification. The migrated and invaded SW13 and NCI-H295R cells were counted in three random fields per well. The experiments were performed in a triplicate manner.

**Separation of polymerized and depolymerized tubulin**

In brief, SW13 (3 × 10^5^) and NCI-H295R (5 × 10^5^) cells were seeded in 6-well tissue culture plates. The next day, cells were treated with OTS167, RGB-286638, a combination of OTS167 and RGB-286638, and vehicle control for 24 hours and 48 hours for SW13 and NCI-H295R, respectively. After treatment, the cells were washed with PBS and harvested with hypotonic lysis buffer (1 mM MgCl_2_, 2 mM EGTA, 0.5% NP-40, and 20 mM Tris HCL [pH 6.8]) containing protease inhibitors. The samples were centrifuged at 13 000*g* for 10 minutes to separate polymerized (P) from soluble (S) tubulin. The supernatant containing the depolymerized (S) tubulin was transferred to a new tube. The pellet containing P tubulin was resuspended in an equal amount of hypotonic lysis buffer. Equal amounts of protein were loaded on sodium dodecyl sulfate polyacrylamide gel electrophoresis (SDS-PAGE) gels and analyzed by Western blotting with anti–α-tubulin and GAPDH antibodies.
**In vivo study of OTS167 and RGB-286638 in mice with human ACC xenografts**

Mice were maintained according to NIH Animal Research Advisory Committee guidelines. A total of 5 × 10^6^ NCI-H295R cells with luciferase reporter were injected into each flank of a Nuþ/Nuþ mouse (two xenografts per mouse). After 21 days, the mice were randomized into four groups by the treatment: Group 1, 0.1% DMSO as vehicle control; Group 2, daily (Monday–Friday) OTS167 (10 mg/kg) via intraperitoneal injection; Group 3, RGB-286638 (20 mg/kg) using an intravenous injection via tail vein three times (Monday, Wednesday, Friday) weekly for two weeks, followed by RGB-286638 drugs (6 mg/100 μL) loaded into ALZET pumps with a 0.25 μL/hour delivery rate (Model 1002, Alzet, Cupertino, CA, USA); Group 4, the combination of OTS167 (10 mg/kg) and RGB-286638 (20 mg/kg), following the drug administration protocols of Groups 2 and 3. Treatment continued for five weeks. Mice received daily health monitoring, and their weight was recorded weekly.

We performed a live bioluminescence imaging study under general anesthesia to quantitate tumor burden using the Xenogen IVIS Spectrum in vivo imaging system (PerkinElmer, Shelton, CT, USA). The signal intensity was quantified as the sum of all detected photon counts within a region of interest. We injected 5 mg of luciferin/mouse intraperitoneally 10 minutes prior to the live-imaging study. Next, the animals were anesthetized in a plastic chamber filled with a 2.5% isoflurane/oxygen/air mixture. This was maintained using a nose-cone delivery system during imaging. The tumor volume using caliper measurement was calculated using formula:Volume ( V) =1/2 (Length x Width ^2^). After five weeks of treatments, all mice were euthanized by CO_2_ inhalation. The ACC xenografts were then removed, flash-frozen in liquid nitrogen, and formalin-fixed. The formalin-fixed xenografts were processed into paraffin blocks for hematoxylin and eosin stain and immunostaining. The terminal blood collection was performed by a cardiac puncher. The blood samples were spun at 1,500*g* for 10 minutes to collect serum for further analysis.

### Statistical analysis

### We analyzed the gene expression profiling data (GSE33371, GSE12368, GSE90713) using embedded interactive statistical software (GEO2R). The *p* values were adjusted for false discovery rate using Benjamini-Hochberg methods (4). The data were presented in a box-plot with a median in the box that represented the 25th and 75th percentiles of data. Error bars demonstrated minimum and maximum values within 95% of data. The associations between the mRNA expressions and survival variables (overall and recurrence-free survival) were assessed using the Kaplan-Meier estimator with the Logrank (Mantel-Cox) test (5). We used Cox proportional hazard regression in the multivariate analysis to identify independent variables associated with the survival variables. We used the analysis of variance (ANOVA) with posthoc tests to compare the mRNA expression and in vivo luciferase activity between groups. If interactions were found, pairwise comparisons between group levels were calculated with the Bonferroni correction for multiple testing. Student’s t*-*test was used to compare the mean between groups that normally distributed. The Mann–Whitney *U* test was used to compare continuous variables that were not normally distributed. A two-tailed *p* value less than 0.05 was considered statistically significant. Statistical analyses were performed using SPSS version 25.0 for Windows (SPSS, Inc., Chicago, IL, USA) and GraphPad Prism 8 software (GraphPad Software, La Jolla, CA, USA).

### References

1. Martinez NJ, Rai G, Yasgar A, Lea WA, Sun H, Wang Y, et al. A High-Throughput Screen Identifies 2,9-Diazaspiro[5.5]Undecanes as Inducers of the Endoplasmic Reticulum Stress Response with Cytotoxic Activity in 3D Glioma Cell Models. *PLoS One.* 2016;11(8):e0161486.

2. Mathews Griner LA, Guha R, Shinn P, Young RM, Keller JM, Liu D, et al. High-throughput combinatorial screening identifies drugs that cooperate with ibrutinib to kill activated B-cell-like diffuse large B-cell lymphoma cells. *Proc Natl Acad Sci U S A.* 2014;111(6):2349-54.

3. Nilubol N, Boufraqech M, Zhang L, Gaskins K, Shen M, Zhang YQ, et al. Synergistic combination of flavopiridol and carfilzomib targets commonly dysregulated pathways in adrenocortical carcinoma and has biomarkers of response. *Oncotarget.* 2018;9(68):33030-42.

4. Sun L, Craiu RV, Paterson AD, and Bull SB. Stratified false discovery control for large-scale hypothesis testing with application to genome-wide association studies. *Genet Epidemiol.* 2006;30(6):519-30.

5. Mantel N. Evaluation of survival data and two new rank order statistics arising in its consideration. *Cancer Chemother Rep.* 1966;50(3):163-70.
