# Supplementary material for: Preclinical assessment of synergistic efficacy of MELK and CDK inhibitors in adrenocortical cancer
Source: J Exp Clin Cancer Res. 2022 Sep 23;41:282. doi: 10.1186/s13046-022-02464-5 (PMC9502945; doi:10.1186/s13046-022-02464-5)
Supplement: Supplementary file 2 — Additional file 2: Supplementary Figure 1. CDKs and cyclin molecules are overexpressed in ACC: A mRNA expression of CDK1 and CDK2 (NC vs. ACC) from the publicly available data set GSE90713 (NC = 5, ACC = 57) (p < 0.05). B mRNA expression of CCNA2, C mRNA expression of CCNB1, D mRNA expression of CCNB2, and E mRNA expression of CCNE2, from the GSE33371, GSE12638, and GSE90713 data sets. Supplementary Figure 2. Cyclin molecule overexpression correlates with poor prognosis in ACC: A Kaplan-Meier survival curve representing the OS in the TCGA ACC cohort with low and high expression of CCNA2, CCNB1, and CCNB2. B Kaplan-Meier survival curve representing the overall DFS in the TCGA ACC cohort with low and high expression of CCNA2, CCNB1, and CCNB2. C, D Differential mRNA expression of CCNA2, CCNB1, and CCNB2 by over all stages and T stage in the TCGA ACC cohort. All data were analyzed by two-tailed unpaired Student’s t-test or one-way ANOVA. Supplementary Figure 3. Cyclin E2 is associated with poor prognosis of patients with ACC: A, B Kaplan-Meier survival curve representing the overall and DFS in the TCGA ACC cohort with high and low CCNE2expression. High CCNE2 expression (red); low CCNE2 expression (blue) (*p < 0.01). C CCNE2 expression by over all stage and T stage in the TCGA ACC cohort (*p < 0.05). Supplementary Figure 4. Cleaved Caspase 3 expression in SW13 and NCI-H295R cells treated with OTS167, RGB-286638, and OTS167 and RGB-286638 combined for 24 and 48 hours respectively. Cells were stain with cleaved caspase 3 conjugated with Alexa Fluor® 555 Conjugate (Red). Actin filaments and nuclei were labeled with Alexa Fluor 488 phalloidin (green) and DAPI respectively. The images were taken under confocal microscopsy (Zeiss) at 63X. Supplementary Figure 5. FOXM1 is overexpressed in advanced tumor stage and correlated with poor prognosis of ACC patient: A mRNA expression of FOXM1 in the publicly available data sets GSE33371 (NC vs. ACC, ACA vs. ACC) (p < 0.001) and GSE12 [file 13046_2022_2464_MOESM2_ESM.docx]

**Supplementary Figure 1**

**
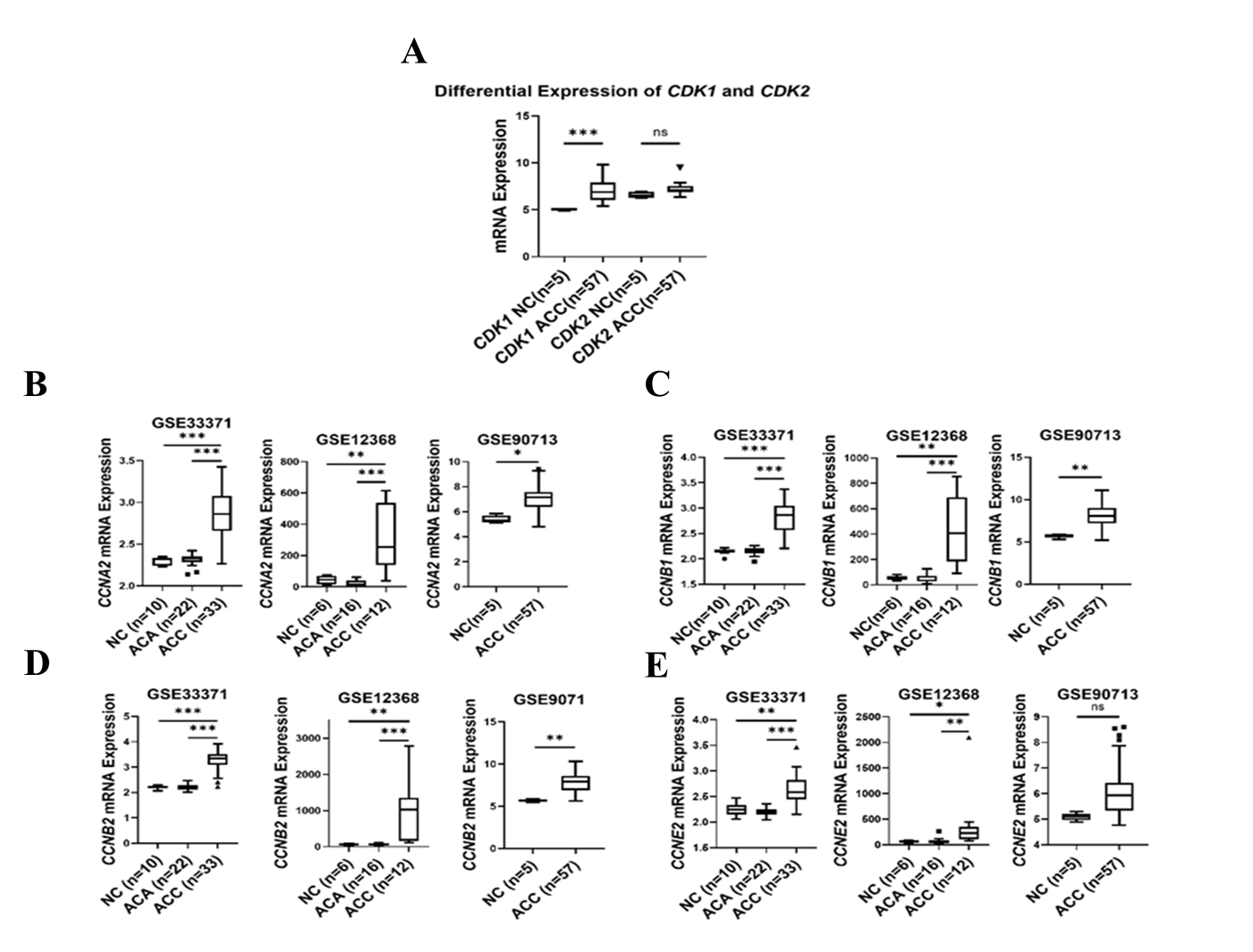
**

**Supplementary Figure 2**

**
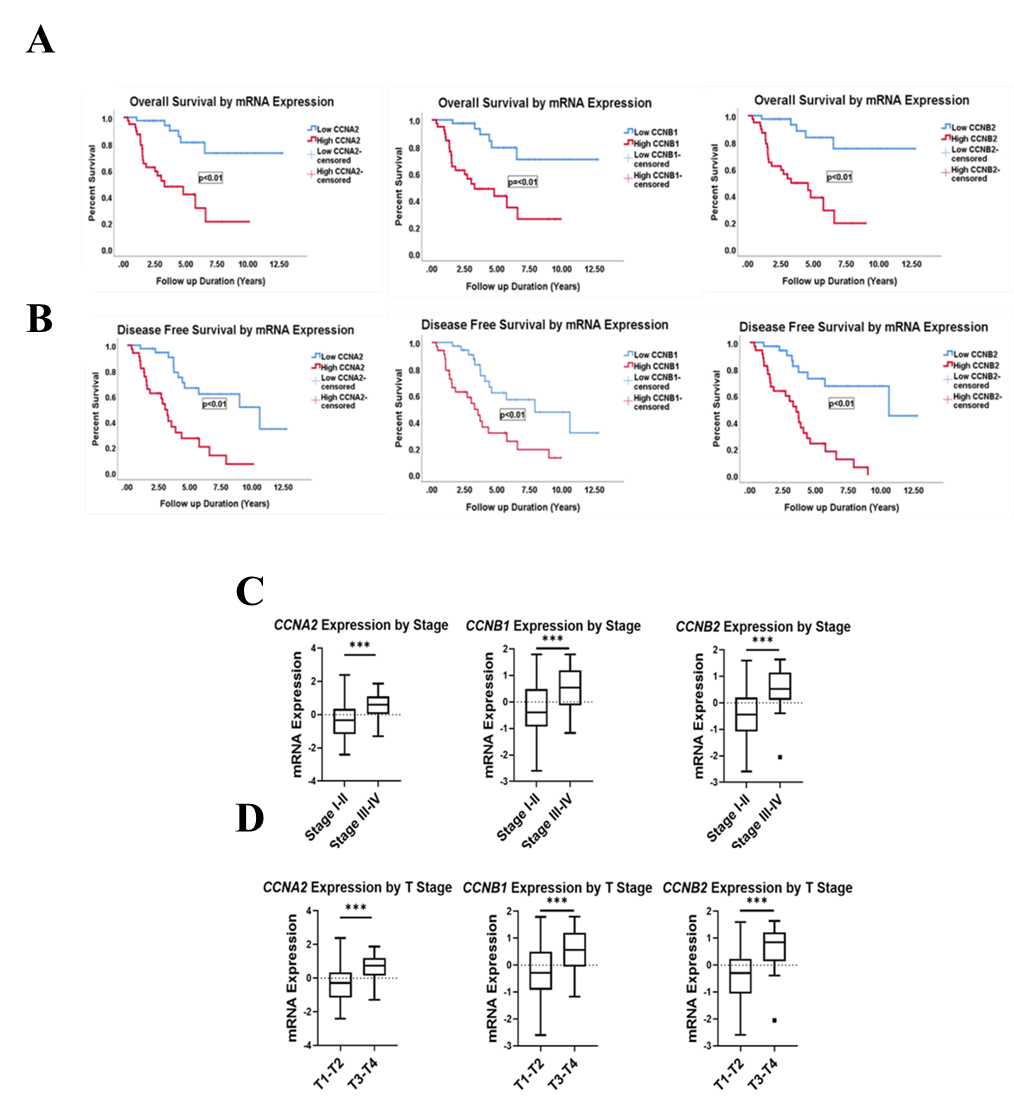
**

**Supplementary Figure 3**

**
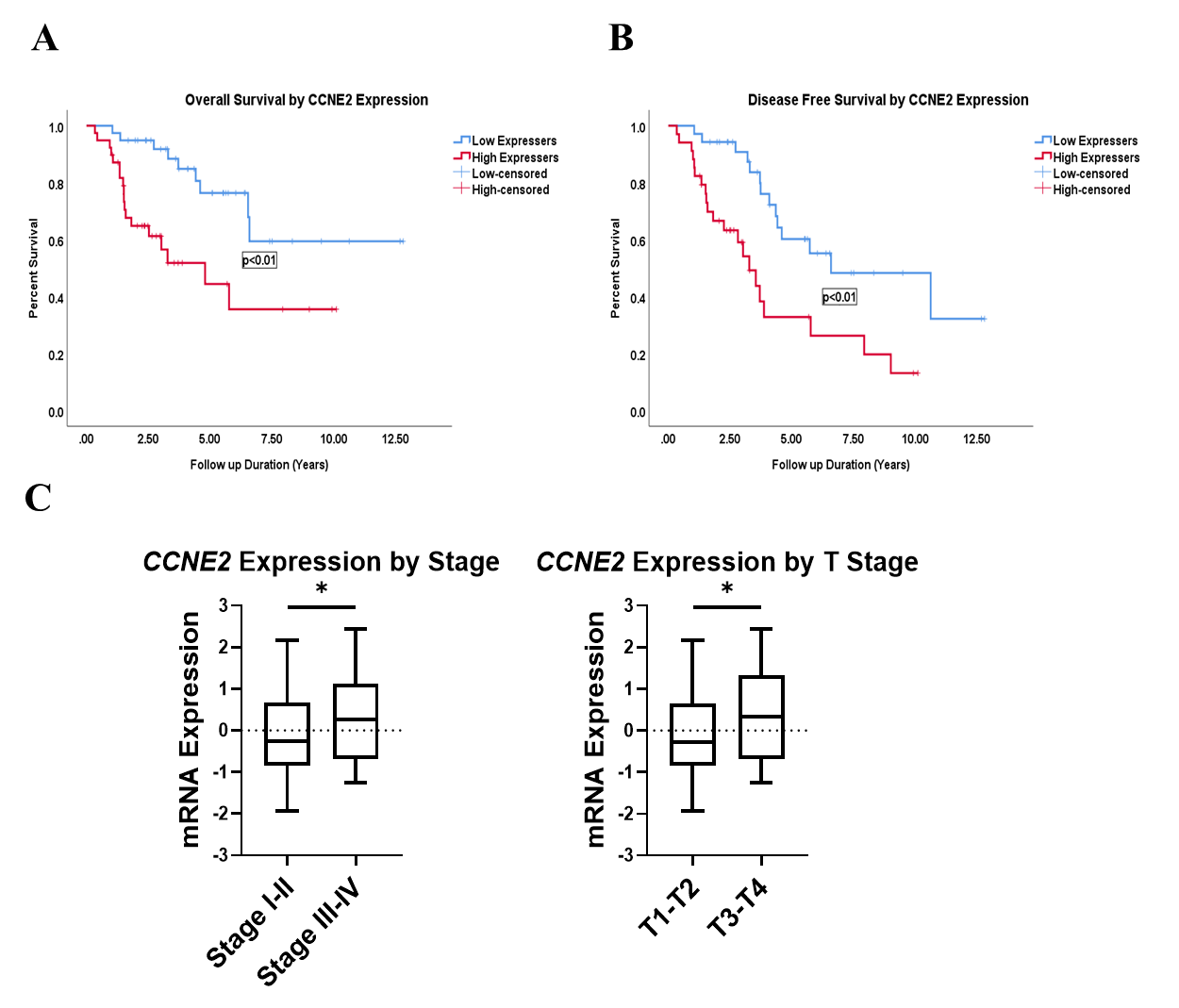
**

**Supplementary Figure 4**

**
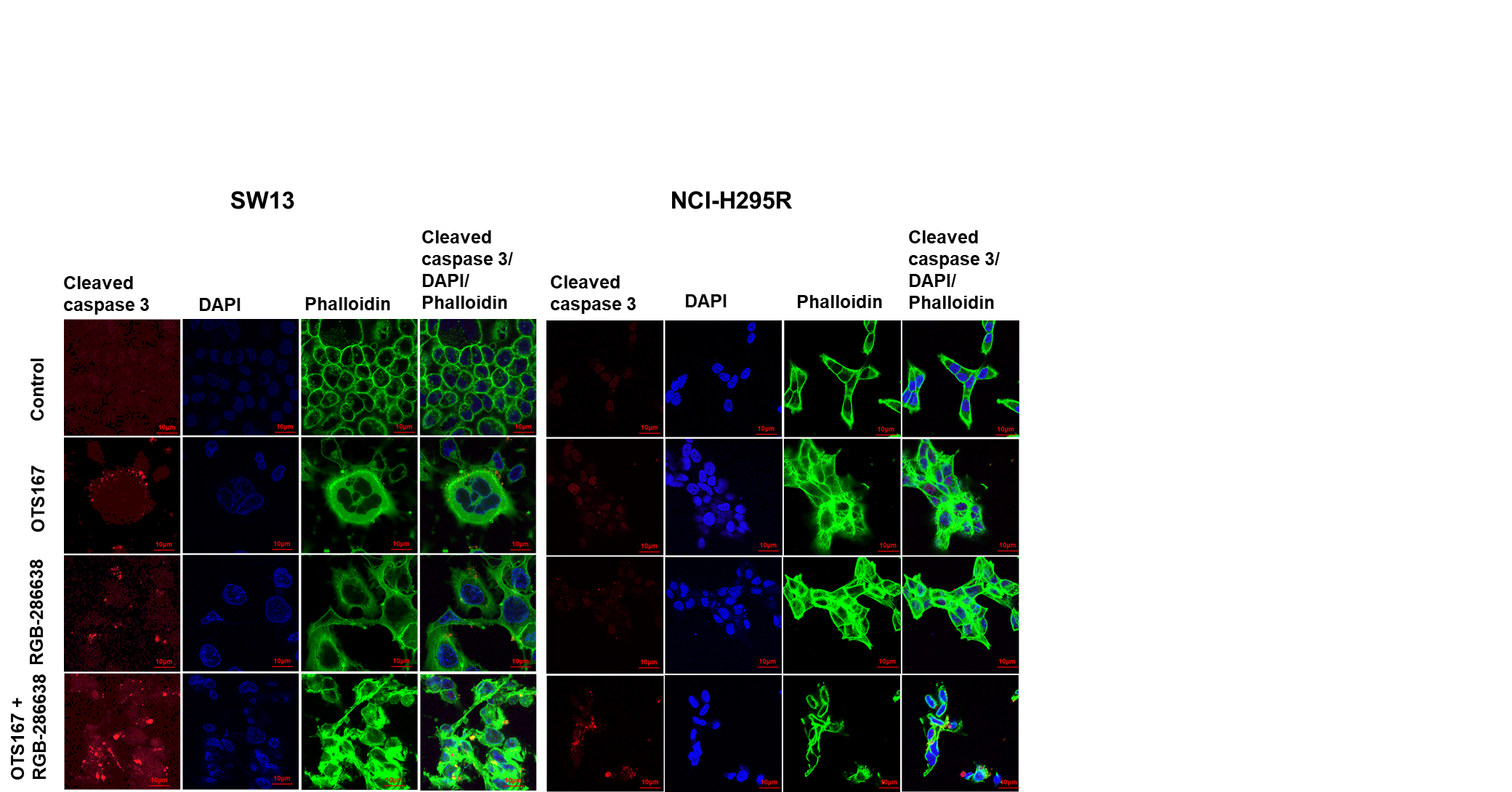
**

**Supplementary Figure 5**

**
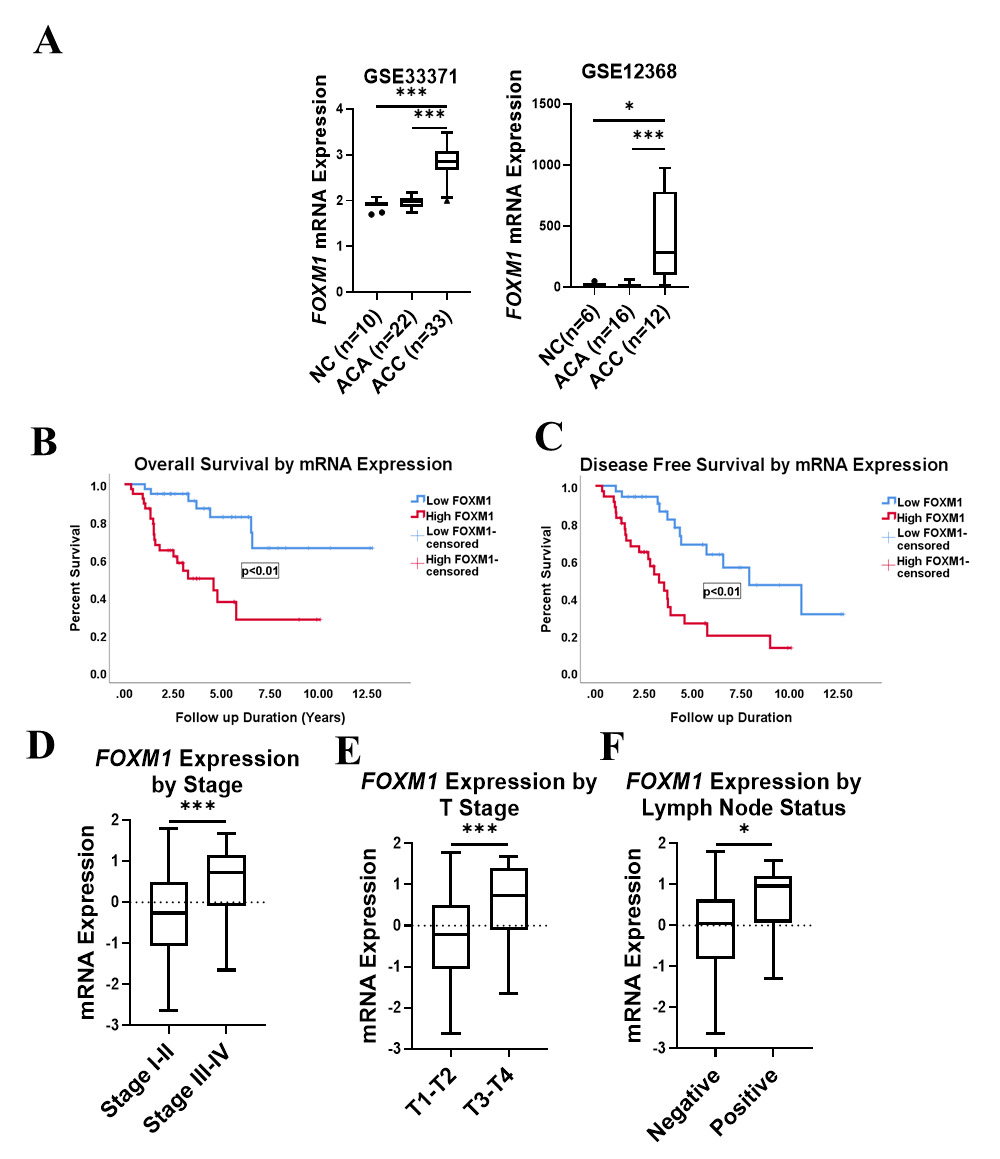
**

**Supplementary Figure 6**

**
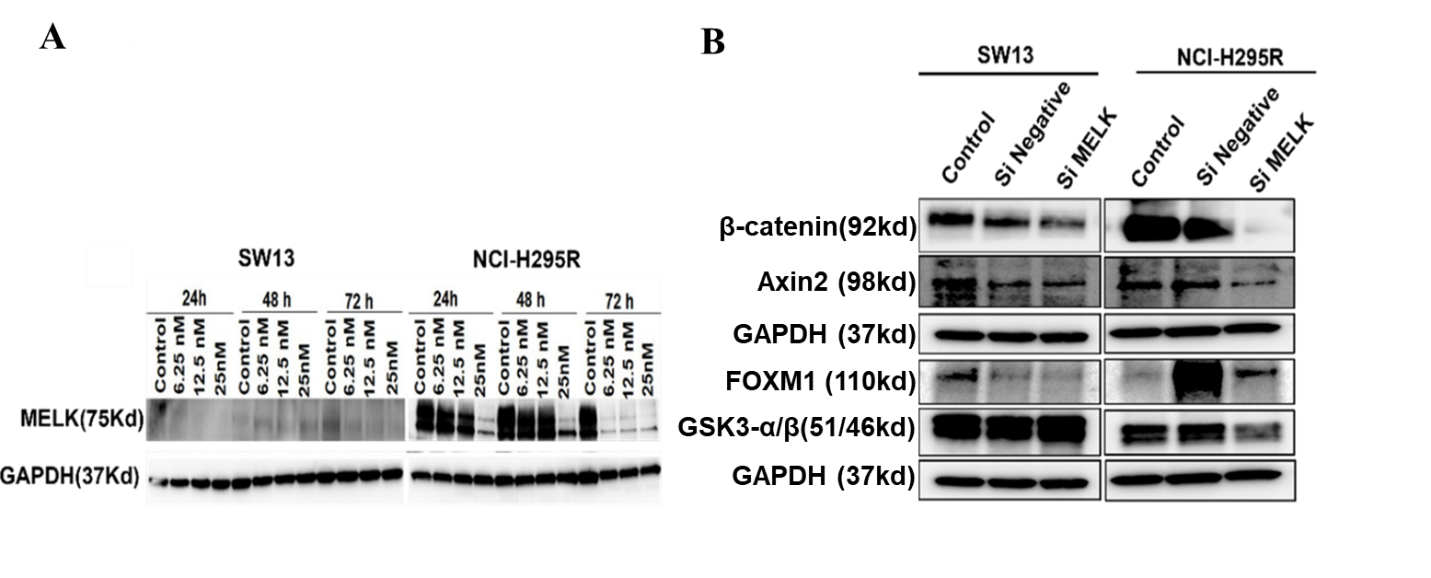
**
