# Supplementary material for: Preclinical assessment of synergistic efficacy of MELK and CDK inhibitors in adrenocortical cancer
Source: J Exp Clin Cancer Res. 2022 Sep 23;41:282. doi: 10.1186/s13046-022-02464-5 (PMC9502945; doi:10.1186/s13046-022-02464-5)
Supplement: Supplementary file 3 — Additionalfile 3: Supplementary Table 1. List of 30 selected drugs for combination drug screening in ACC cell lines. Supplementary Table 2. Correlations between mRNA expression of CDKs and cyclin molecules with MKI67 in human ACC from the TCGA cohort Supplementary Table 3. Correlations between mRNA expression of CDKs and cyclin molecules with MELK in human ACC samples from the TCGA cohort. Supplementary Table 4. OTS167 and RGB-286638 combination shows synergistic activity in ACC cells. The combination index (CI) was calculated using the Chou–Talalay method. The CI is determined by the following range: CI < 1, synergistic; CI = 1, additive; CI > 1, antagonistic. Supplementary Table 5. Correlations between mRNA expression of MELK and β-catenin regulatory molecules in human ACC samples from the TCGA cohort Supplementary Table 6. Correlations between mRNA expression of CDKs and cyclin molecules with STMN1 in human ACC samples from the TCGA cohort Supplementary Table 7. List of Antibodies. [file 13046_2022_2464_MOESM3_ESM.docx]

**Supplementary Table 1:** List of 30 selected drugs for combination drug screening in ACC cell lines.

| **Drugs Name** | **Primary MOA** | **AC50 (μM)** | |
| --- | --- | --- | --- |
|  |  | **NCI-H295R** | **SW13** |
| Sepantronium bromide | Survivin Inhibitor | 0.0037 | 0.0029 |
| Delanzomib | Proteasome Inhibitor | 0.0906 | 0.1280 |
| Bortezomib | Proteasome Inhibitor | 0.0523 | 0.0930 |
| GSK-461364A | Polo-Like Kinase-1 Inhibitor | 0.0930 | 0.1171 |
| Oxibendazole | DNA Polymerase Inhibitor | 0.4660 | 0.4660 |
| HSP-990 | Heat Shock Protein 90 Inhibitor | 0.1855 | 0.3702 |
| Fenbendazole | Antiparasitic Agent | 0.4153 | 0.4153 |
| Flubendazole | Antiparasitic Agent | 0.4660 | 0.4153 |
| Indibulin | Tubulin Polymerization Inhibitor | 0.4660 | 0.6583 |
| Briciclib | Polo-Like Kinase-1 Inhibitor | 0.0676 | 0.0302 |
| Demecolcine | Tubulin Polymerization Inhibitor | 0.0930 | 0.1171 |
| Ispinesib | Kinesin-Like Spindle Protein Inhibitor | 0.0234 | 0.0186 |
| Litronesib | Kinesin-Like Spindle Protein KIF11 Inhibitor | 0.0052 | 0.0047 |
| OTS167 | MELK Inhibitor | 0.1342 | 0.2689 |
| RGB-286638 | CDK/Cyclin Complex Inhibitor | 0.5867 | 0.7386 |
| ARRY-520 | Kinesin-Like Spindle Protein Inhibitor | 0.0059 | 0.0052 |
| PIK-75 | PI3K Inhibitor | 0.3299 | 0.4660 |
| SB-743921 | Kinesin-Like Spindle Protein Inhibitor | 0.0744 | 0.0066 |
| Trabectedin | DNA-Damaging Drug | 0.0477 | 0.0066 |
| Emetine | Antiprotozoal Agent | 0.4153 | 0.7386 |
| Lurbinectedin | RNA Polymerase Inhibitor | 0.0093 | 0.0093 |
| TP-0903 | AXL Kinase Inhibitor | 0.4262 | 0.6021 |
| SJG-136 | DNA-Intercalating Drugs | 0.0208 | 0.0147 |
| Staurosporine | CDK1/2 Inhibitor | 0.2621 | 0.5229 |
| NVP- AUY922 | Heat Shock Protein 90 (hsp90) Inhibitor | 0.1043 | 0.3299 |
| Echinomycin | HIF-1 Inhibitor | 0.0059 | 0.0052 |
| Ganetespib (STA-9090) | Heat Shock Protein 90 (hsp90) Inhibitor | 0.1043 | 0.2336 |
| MLN-7243 | Ubiquitin-Activating Enzyme E1 Inhibitor | 0.0739 | 0.0829 |
| Daporinad or APO866 | NAMPT Inhibitor | 0.0147 | 0.0208 |
| 7-Hydroxystaurosporine | CDK1/2/4/6 | 0.5366 | 1.2012 |

**Supplementary Table 2:** Correlations between mRNA expression of *CDK*s and cyclin molecules with *MKI67* in human ACC from the TCGA cohort.

| Genes | Spearman Correlation Coefficient Value | Corresponding “*p”* Value |
| --- | --- | --- |
| *MKI67* vs. *CDK1* | 0.95 | 5.69e-40 |
| *MKI67* vs. *CDK2* | 0.65 | 1.22e-10 |
| *MKI67* vs. *CCNA2* | 0.91 | 1.71e-30 |
| *MKI67* vs. *CCNB1* | 0.83 | 2.04e-20 |
| *MKI67* vs. *CCNB2* | 0.85 | 2.59e-22 |
| *MKI67* vs. *CCNE2* | 0.78 | 5.59e-17 |

**Supplementary Table 3:** Correlations between mRNA expression of *CDK*s and cyclin molecules with *MELK* in human ACC samples from the TCGA cohort.

| Genes | Spearman Correlation Coefficient Value | Corresponding “*p”* Value |
| --- | --- | --- |
| *MELK* vs. *CDK1* | 0.89 | 6.11e-28 |
| *MELK* vs. *CDK2* | 0.64 | 4.89e-10 |
| *MELK* vs. *CCNA2* | 0.87 | 4.92e-24 |
| *MELK* vs. *CCNB1* | 0.86 | 1.06e-23 |
| *MELK* vs. *CCNB2* | 0.87 | 1.30e-24 |
| *MELK* vs. *CCNE2* | 0.74 | 1.48e-14 |

**Supplementary Table 4:** OTS167 and RGB-286638 combination shows synergistic activity in ACC cells. The combination index (CI) was calculated using the Chou–Talalay method. The CI is determined by the following range: CI < 1, synergistic; CI = 1, additive; CI > 1, antagonistic.

| Time Points (Days) | SW13 | | NCI-H295R | |
| --- | --- | --- | --- | --- |
|  | OTS167 (6.25 nM)  + RGB-286638 (12.5 nM) | OTS167 (12.5 nM)  + RGB-286638 (25 nM) | OTS167 (6.25 nM) + RGB-286638 (6.25 nM) | OTS167 (12.5 nM) + RGB-286638 (12.5 nM) |
| Day 1 | 0.67 | 1.12 | 0.61 | 1.16 |
| Day 2 | 0.2257 | 1.1291 | 0.43 | 0.82 |
| Day 3 | 0.2815 | 0.2758 | 0.39 | 0.49 |
| Day 4 | 0.6858 | 0.54404 | 0.50 | 0.65 |
| Day 5 | 0.4860 | 0.4290 | 0.31 | 0.15 |

**Supplementary Table 5:** Correlations between mRNA expression of *MELK* and β-catenin regulatory molecules in human ACC samples from the TCGA cohort.

| Genes | Spearman Correlation Coefficient Value | Corresponding “*p*” Value |
| --- | --- | --- |
| *MELK* vs. *FOXM1* | 0.85 | 1.29e-22 |
| *CTNNB1* vs. *AXIN2* | 0.54 | 3.17e-7 |
| *AXIN2* vs. *GSK3B* | 0.34 | 2.302 e-3 |
| *CTNNB1* vs. *GSK3B* | 0.31 | 5.327 e-3 |

**Supplementary Table 6:** Correlations between mRNA expression of *CDKs* and cyclin molecules with *STMN1* in human ACC samples from the TCGA cohort.

| Genes | Spearman Correlation Coefficient Value | Corresponding “*p*” Value |
| --- | --- | --- |
| *STNN1* vs. *CDK1* | 0.87 | 7.03e-25 |
| *STMN1* vs. *CDK2* | 0.63 | 9.45e-10 |
| *STMN1* vs. *CCNA2* | 0.80 | 5.36e-18 |
| *STMN1* vs. *CCNB1* | 0.79 | 8.01e-17 |
| *STMN1* vs. *CCNB2* | 0.82 | 4.78e-19 |
| *STMN1* vs. *CCNE2* | 0.62 | 1.62e-9 |
| *STMN1 vs FOXM1* | 0.75 | 3.65e-15 |
| *STMN1 vs MELK* | 0.83 | 8.87e-21 |

**Supplementary Table 7:** List of Antibodies.

| Antibodies | Dilution/Concentration | Company & Cat. No. | Methods |
| --- | --- | --- | --- |
| MELK | 1:200 | Abcam, ab129373 | IHC |
| Ki67 (Human) | 1:100 | CST, 9027S | IHC |
| Ki67 (Mouse) | 1:100 | CST, 9129S | IHC |
| MELK | 1:1000 | Abcam, ab108529 | WB |
| Cyclin B1 | 1:1000 | CST, 12231S | WB |
| Cyclin B2 | 1:1000 | Abcam, ab185622 | WB |
| Cyclin A2 | 1:1000 | CST, 91500S | WB |
| Cleaved Caspase 3 | 1:1000 (WB), 1:200 (IHC) | CST, 9661S | WB |
| Cleaved Caspase 3 | 1:50 | CST 9604S | IF |
| α-tubulin | 1:1000 | CST 3873S | IF |
| Fibronectin | 0.25 μg/ml | BD Transduction Laboratories™, 610078 | WB |
| N-Cadherin | 1:1000 | CST, 13116S | WB |
| Vimentin | 1:1000 (WB), 1:100 (IHC) | CST, 5741S | WB, IHC |
| β-Catenin | 1:1000 (WB),  1:100 (IF),  1:100(IHC) | CST, 8480S | WB, IF, IHC |
| Axin2 | 1:1000 | CST, 2151S | WB |
| α-tubulin |  |  |  |
| GSK3-α/β | 1:1000 | CST, 5676T | WB |
| FOXM1 | 1:1000 | CST, 5436S | WB |
| Stathmin1 | 1:1000 | CST, 3352S | WB |
| phospho-Stathmin1 | 1:1000 | CST, 4191S | WB |
| GADPH | 1:3000 | CST, 5174S | WB |
| Histone H3 | 1:3000 | CST, 4499S | WB |
| Anti-Rabbit HRP | 1:2000 | CST, 7074S | WB |
| Anti-Mouse HRP | 1:200 | CST, 7076S | WB |
| Alexa Fluor™ Goat Anti-Rabbit 594 | 1:100 | Thermo Fisher Scientific, A32740 | IF |
| Alexa Fluor™ Goat Anti-Mouse 488 | 1:200 | Thermo Fisher Scientific  A32723 | IF |

**Abbreviations:** CST, Cell Signaling Technology; IHC, immunohistochemistry; IF, immunofluorescence; WB, Western blot.
